# Entropy-Based Model for MiRNA Isoform Analysis

**DOI:** 10.1371/journal.pone.0118856

**Published:** 2015-03-18

**Authors:** Shengqin Wang, Jing Tu, Lei Wang, Zuhong Lu

**Affiliations:** 1 State Key Lab of Bioelectronics, School of Biological Science and Medical Engineering, Southeast University, Nanjing, 210096, China; 2 Zhejiang Provincial Key Laboratory for Water Environment and Marine Biological Resources Protection, College of Life and Environmental Science, Wenzhou University, Wenzhou, 325035, China; 3 Department of Biomedical Engineering, College of Engineering, Peking University, Beijing, 100781, China; The John Curtin School of Medical Research, AUSTRALIA

## Abstract

MiRNAs have been widely studied due to their important post-transcriptional regulatory roles in gene expression. Many reports have demonstrated the evidence of miRNA isoform products (isomiRs) in high-throughput small RNA sequencing data. However, the biological function involved in these molecules is still not well investigated. Here, we developed a Shannon entropy-based model to estimate isomiR expression profiles of high-throughput small RNA sequencing data extracted from miRBase webserver. By using the Kolmogorov-Smirnov statistical test (KS test), we demonstrated that the 5p and 3p miRNAs present more variants than the single arm miRNAs. We also found that the isomiR variant, except the 3’ isomiR variant, is strongly correlated with Minimum Free Energy (MFE) of pre-miRNA, suggesting the intrinsic feature of pre-miRNA should be one of the important factors for the miRNA regulation. The functional enrichment analysis showed that the miRNAs with high variation, particularly the 5’ end variation, are enriched in a set of critical functions, supporting these molecules should not be randomly produced. Our results provide a probabilistic framework for miRNA isoforms analysis, and give functional insights into pre-miRNA processing.

## Introduction

MiRNAs are ~22 nt endogenous small non-coding RNAs, mediating the translation repression or trigger degradation by paring with target mRNAs in post-translational regulation to control gene expression [1,2]. Advances in next-generation sequencing (NGS) technology are giving rise to a fast accumulation of known miRNAs. In the lasted miRBase version, the human genome encodes for over 1,500 miRNAs [3].

Typically, a mature miRNA commences from the genome as a primary miRNA transcript (pri-miRNA) via RNA polymerase II-mediated transcription. Together with DGCR8, the nuclear RNase III-type protein Drosha cleaves the pri-miRNA to release the precursor miRNA (pre-miRNA), a hairpin-like secondary structure. With the exportin 5-dependent pathway, the pre-miRNA is then exported to the cytoplasm, where it is processed into a short double-stranded RNA (dsRNA) duplex by the enzyme Dicer [4,5]. One or both strands of the duplex may serve as the functional mature miRNA, and anneal to target mRNA that have complementary target sequence with the guide of the RNA-induced silencing complex (RISC) [5,6]. The imprecise precursor cropping or dicing can change the Drosha and Dicer cleavage sites and generate miRNA isoform products, which make variations in their 5’ and/or 3’ end positions compared with canonical miRNAs [7].

Many high-throughput small RNA sequencing projects have demonstrated the existence of isomiR variants [8–11]. The frequency of variations at same sites is seen repeatedly and unlikely attribute to degradation or sequencing error, and some of them have been proved to play an important biological role in the control of miRNA-mediated gene expression [12–16]. Variant in the 5’ end position of miRNA is supposed to alter the seed region, which is supposed to be very important for target recognition [17–19], thereby reshuffling the target region and affecting the related biological pathway [20–22]. And adding specific nucleotides to the 3’ end can modify the stability of miRNA and/or the efficiency of target repression[23–25].

To our knowledge, the isomiR profile can be attributable to three main factors: Drosha and Dicer cleavage, nucleotide addition, and nucleotide substitution. The template nucleotide addition can be the result of the imprecise cleavage by Drosha and Dicer, which has been reported to be more frequent than the non-template nucleotide addition [26,27]. The non-template nucleotide addition can be originated in nucleotide addition [23] or nucleotide substitution by post-transcriptional modifications [28]. Most of non-template nucleotide additions are located at 3’ end of miRNAs, and the frequency of them is quite low based on the pervious transcriptome data analysis [29].

Despite the distribution of isomiRs is unlikely to be random, the biological relevance of these molecules has been overlooked in previous studies[7]. Here, we developed a Shannon entropy-based model to measure the isomiR expression profiles from high-throughput small RNA sequencing data, and to find the candidate functional role of these molecules.

## Materials and Methods

### Data sources

We fetched the high-throughput small RNA sequencing data for multiple alignment format employed in miRBase webserver [3], including 81 Homo sapiens related experiments collected from five recently published papers [30–34]. These experiments included miRNAs from different developmental stages of different tissues and cell lines, and the multiple alignment data pooled these miRNAs together. Corresponding pre-miRNAs and their Minimum Free Energy (MFE) information were also retrieved. Since too few sequences will result in a systematic underestimation of isomiR variants, as well as too many sequences may be contributed by PCR amplification bias, our analysis only included miRNAs with number of sequences more than 50 and less than 10000 (S1 Fig.).

### Shannon entropy calculation

At first, we defined the isomiRs as sequences that matched the known pre-miRNA in the canonical mature miRNA region ± 4 nt. To characterize the isomiR variants of a given miRNA, in the case of multiple alignments of miRNA sequences, we defined *MIH*, MiRNA Isoform entropy (abbreviated H), as the average of Shannon entropies of the observed symbol distribution for each site in the related region:

$$MIH=-\frac{1}{L}\sum_{i}^{L}\sum_{j}^{N}p_{j}^{i}log_{2}p_{j}^{i}$$ (1)

**Fig 1:**
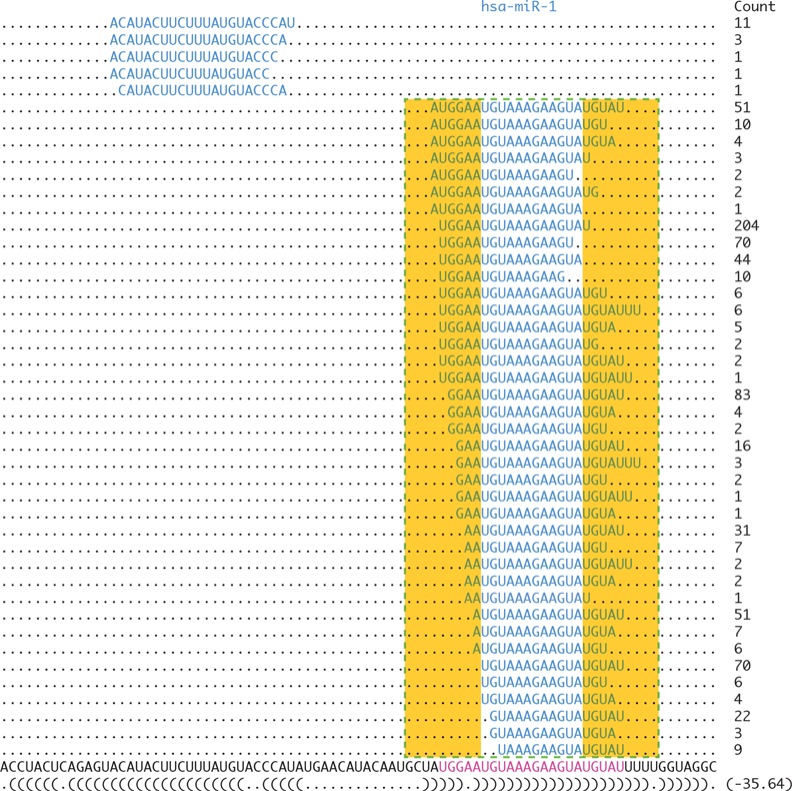
Region selection for *MIH* calculation. Sequences located in the dotted frame (canonical mature miRNA region ± 4 nt) are accepted for *MIH* calculation. Bases located in the left rectangle are used for *MIH*5 calculation, and those located in the right rectangle are used for *MIH*3 calculation (including the gap character “-”).

Here, *p* ^*i*^_*j*_ is the observed frequency which calculated as the frequency of the character j at particular sequence position i divided by the number of sequences in the alignment, and N is the number of distinct symbols for the given sequence type (five for RNA: A, U, G, C, and gap character “-”). For *MIH* calculation, L is equivalent to the length of canonical mature miRNA + 8, including the upstream 4 nt and downstream 4 nt. Since the variations differ greatly between 5’ ends and the 3’ ends, we calculated *MIH*5 for 5’ end variations and *MIH*3 for 3’ end variations, respectively. For both of them, L is equal to 9, including the end site and flanking region (± 4 nt). For example, as shown in Fig. 1, the *MIH* of has-miR-1 is defined as the average of entropies of the symbol distribution for all positions located in the green dotted frame, and corresponding *MIH*5 and *MIH*3 can be calculated from the left and right yellow rectangle region.

### Functional enrichment analysis

Here, we took TAM tool to perform enrichment analysis to get an insight into the functional role of the miRNAs with high isoform entropy values [35]. The TAM collected miRNAs and their related functions reported in the publications. After removing redundancy, the corresponding high isoform entropy precursors were used to perform functional enrichment analysis, and all precursors with characterized isomiR variants were selected as the reference set. We also used the DAVID Bioinformatics Tools[36,37] to estimate the pathway enrichment of the experimentally validated miRNA targets that were supported by assay or Western blot from miRTarBase[38] and of the predicted miRNA targets from TargetScan[39], respectively. The Enrichment Thresholds or EASE was set as 0.05.

## Results

### Summary of miRNA isoform entorpy values

**Fig 2:**
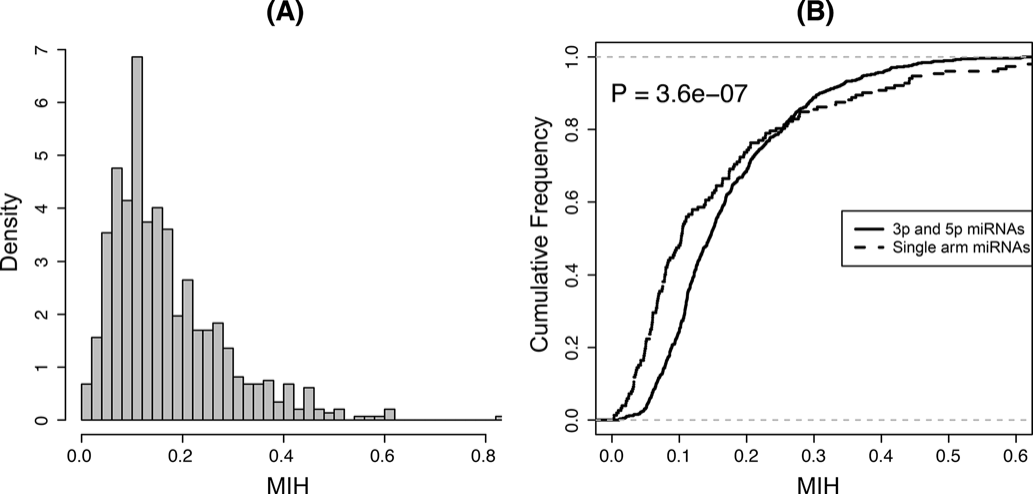
Distribution of *MIH* values. (A) *MIH* distribution of all mature miRNAs after filter. (B) KS-test comparison cumulative fraction plot. Solid line indicates the cumulative distribution of mature miRNA *MIH* values from both 5’ and 3’ arms of the hairpin precursors (the 3p and 5p miRNAs). Dot line shows the cumulative distribution of mature miRNA *MIH* values from only single arms (the single arm miRNAs).

In this study, we calculated *MIH* values using the small RNA sequences extracted from miRBase website. After discarding miRNAs with the very large or very small numbers, we collected 736 mature miRNAs from 545 precursors with varied *MIH* range from 0.003 to 1.096 (S1 Table), which show the presence of sequence heterogeneity for all the analyzed miRNAs, supporting the complexity of Drosha and Dicer processing in the pre-miRNA transcription [40,41]. High *MIH* value demonstrates blurred patterns and broad diversity of miRNA processing, while low *MIH* value means the expression of isomiRs is dominant on some mature miRNA sequences and great uniformity along the pre-miRNA. Thus, *MIH* is suitable to compare the variant profiles across different miRNAs. From density plot Fig. 2A, we found most of *MIH* values are located in a region around 0.1, which means most of the miRNAs have low isoform variations, coinciding with the result of previous study[42]. For example, the MIH score of miR-1 in Fig. 1 is 0.1956 (S1 Table).

These *MIH* values can be divided into two sets by the name of mature miRNAs, one set named with 5p or 3p means both arms of pre-miRNAs can generate mature miRNAs without regarding to the preferred strand (the 5p and 3p miRNAs), and the other set named without 5p or 3p means these miRNAs prefer one arm of pre-miRNAs (the single arm miRNAs). Here, we applied the KS test for determining whether isomiR distribution patterns are different between the 5p and 3p miRNAs and the single arm miRNAs. KS test can be used to check if two sets differ significantly under making no assumption about the distribution of data. Compared with the single arm miRNAs, the 3p and 5p miRNAs have greater *MIH* values (Fig. 2B), which means the 3p and 5p miRNAs have greater isoform variations, demonstrating the entropy of products from pre-miRNA (mature miRNA types) is consistent with the entropy of mature miRNA sequences (isomiR variants).

In order to find the relative contributions of Drosha and Dicer to the isomiR variants, we compared the *MIH5* and *MIH3* values of the paired 5p and 3p miRNAs, respectively (S1 Table. Dependent 2-group Wilcoxon Signed Rank Test). We found that the mean of the 3p miRNA *MIH3* values (Drosha cleavage, mean = 0.321) is less than the mean of the 5p miRNA *MIH3* values (Dicer cleavage, mean = 0.359), and the difference between them is significant (*P* value < 0.01). The mean of the 5p miRNA *MIH5* values (Drosha cleavage, mean = 0.146) is also less than the mean of the 3p miRNA *MIH5* values (Dicer cleavage, mean = 0.151), though the difference between them is not significant (*P* value > 0.1). Our result agreed with the report that Drosha is higher fidelity than Dicer, which is described in a recent study based on the dominant isomiR analysis in 17 mouse samples [27]. Comparing with their study, our analysis only included the miRNAs with high expression level (count >50), which might be one of the proposed reasons why we cannot detect the significant difference between the Drosha cleavage and Dicer cleavage at the 5’ end.

### Correlation analysis

**Fig 3:**
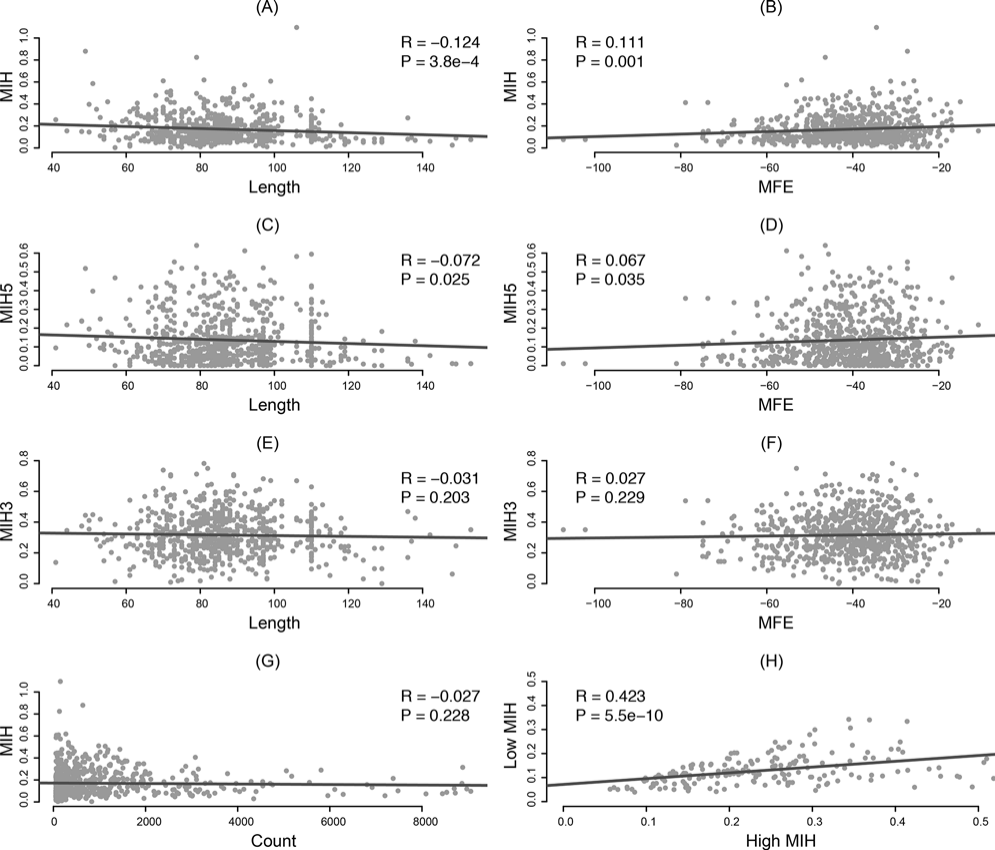
Pearson’s correlation analysis. (A) *MIH* and length of pre-miRNA; (B) *MIH* and MFE; (C) *MIH5* and length of pre-miRNA; (D) *MIH5* and MFE; (E) *MIH3* and length of pre-miRNA; (F) *MIH3* and MFE; (G) *MIH* and count of reads; (H) High *MIH* and low *MIH*. High and low *MIH* values are calculated by comparing the *MIH* values between the 5p and 3p miRNAs from the same precursor.

Here, we investigated the MFE and length of pre-miRNA to figure out if the intrinsic feature contributes to the isomiR expression profiles. By comparing *MIH* and length with MFE extracted from miRBase, we found the MFE and length of pre-miRNAs should have a great contribution to the isomiR variants (Fig. 3A and B). It seems that the thermostability of folding secondary structures can result in the variation in the Drosha and Dicer cleavage sites. Interestingly, the *MIH5* was also detected to be correlated with the MFE and length of pre-miRNA, while the *MIH3* was not (Fig. 3C, D, E and F). We also compared *MIH* with the number of miRNA sequences to check if the abundance of expression level can affect the *MIH* values, whereas they are not significantly correlated (Fig. 3G), which means the sequence depth can not give a reason for the difference of *MIH* values between miRNAs.

Since *MIH* is positively correlated with MFE, the isomiR expression profiles of mature miRNAs transcribed from two arms of pre-miRNAs should be also correlated. For most pre-miRNAs, both arms can generate functional mature miRNAs, and show various isomiR expression profiles [43]. However, different pre-miRNAs show diversity dominant mature miRNAs between 5p arm and 3p arm, and present different *MIH* values between 5p and 3p. Some miRNAs have higher *MIH* at 5p arms and others at 3p arms. In order to verify whether the *MIH* values are consistent between the 5p and 3p miRNAs, here, we classified them into two sets (high *MIH* and low *MIH*) by simply comparing the 5p and 3p *MIH* values with each other, and calculated the relationship between them (Fig. 3H). Our result showed the *MIH* values are well correlated between 5p and 3p arms, indicating the isomiRs derived from the same pre-miRNAs show consistent expression profile, which strongly implies that there could be some similar regulation mechanism controlling miRNA isoform production from two arms. It is also pointed out that not all of 5p and 3p arm miRNAs have consistent isomiR expression profiles, sometime extremely high *MIH* in one arm and extremely low *MIH* in another, which demonstrates there should be other more complexly regulate mechanisms existed.

### Functional enrichment analysis

**Fig 4:**
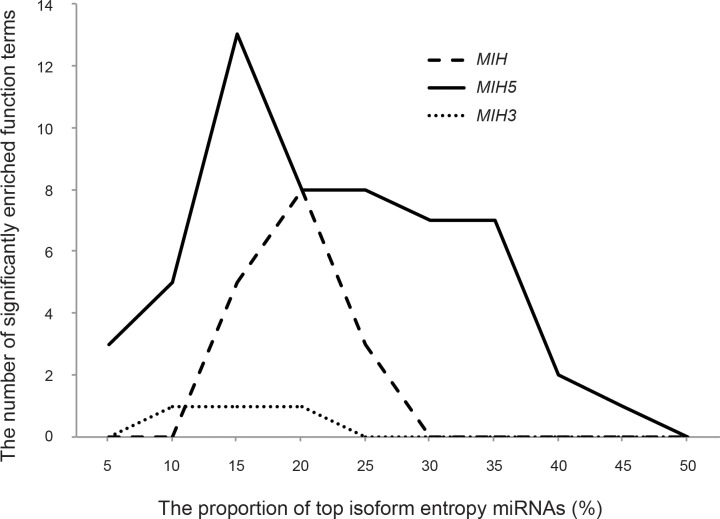
(A) The functional enrichment analysis for miRNA with high isoform entropy values by TAM tool. The X-axis displays the proportion of the top isoform entropy miRNAs. The Y-axis is the number of significantly enriched function terms.

The isomiR expression profiles are non-random, indicating that these sequences could be regulated and therefore functional. In order to assess the biological relevance of these molecules, the TAM tool was utilized to perform functional enrichment analysis for miRNAs with high isoform entropy values. We found the *MHI5* have the greatest significantly enriched function items, while the *MIH3* have little contribution to the enrichment analysis (Fig. 4). Compared with the variations at the 3’ end, the variations at the 5’ end usually have lower values and exhibits higher fidelity, illustrating more biological function repression existed.

The *MIH5* have the largest number of significantly enriched function items when using the top 15% entropy values. After removing redundancy, 103 corresponding precursors were assigned to determine a *P* value for the functional enrichment of the target genes by compared with 545 precursors as a reference set. As shown in Table 1, many miRNAs with high *MIH*5 values are enriched in cancer-related function, such as miRNA tumor suppressors, angiogenesis, cell cycle related, cell differentiation, etc. In order to further check the function of these miRNA, 729 experimentally validated targets extracted from miRTarBase and 1269 conserved target genes predicted by the TargetScan are used for pathway analysis. Of which, 703 experimentally validated targets and 1237 predicted targets are accepted for the DAVID Bioinformatics Tools to estimate the pathway enrichment, respectively. Based on the reported KEGG pathway terms, both of them are also enriched in cancer-related pathway (S2 and S3 Tables).

**Table 1:** Significantly enriched functions of the MiRNAs with the top 15% *MIH5* values.

Function term	Count	*P*-value	FDR
miRNA tumor suppressors	19	3.17E-06	3.56E-04
Cell cycle related	24	2.25E-05	1.44E-03
HIV latency	11	4.50E-05	2.53E-03
Hormones regulation	21	9.14E-05	4.10E-03
Apoptosis	17	5.75E-04	1.99E-02
Angiogenesis	11	8.13E-04	2.43E-02
Akt pathway	9	1.16E-03	2.91E-02
Immune response	16	1.44E-03	3.23E-02
Cell proliferation(Hwang etal BJC2006)	5	2.26E-03	3.76E-02
Folliculogenesis	5	2.26E-03	3.90E-02
Human embryonic stem cell(hESC) regulation	21	2.71E-03	4.32E-02
onco-miRNAs	12	3.43E-03	4.69E-02
Cell proliferation	11	3.70E-03	4.80E-02

## Discussion

In this study, we developed the Shannon entropy-based model *MIH* for the isomiR variant measurement. The variation in the 5’ end and 3’ end leads to a dominating effect on the entropy and gives a *MIH* value. Most of *MIH* values are low, which means the variation expression is still rare. We found that both MFE and length of pre-miRNA are strongly correlated with the variation of isomiRs (Fig. 3A and B), which gives a clue that the intrinsic feature of pre-miRNA should be one of the important factors for the miRNA regulation. The intrinsic feature related miRNA regulation was also found in a recent report that the thermostability of seed binding is strongly correlated with the percentage of seed-pairing target sites[17]. The entropy of miRNA types produced from pre-miRNA should be consistent with the entropy of isomiRs. Then, more types of mature miRNAs are correlated with more isomiR variants, and the 5p and 3p miRNAs usually have larger *MIH* values than the single arm miRNAs (Fig. 2B). In addition, the variations at 5p arms are well correlated with the variations at 3p arms (Fig. 3H).

IsomiR variants, which are not randomly produced by the cleavage of Drosha and Dicer or other mechanisms, should be the characteristic of miRNA expression response under specific condition. Here, we detected the isomiR expression profile at both 5’ and 3’ end by integrating miRNAs under different developmental stages of different tissues and cell lines. The candidate functions of miRNAs with high isoform entropy are enriched in a set of critical functions, strongly implying that there could be important biological role involved, indicating that the complete repertoire of functional miRNAs is likely more complex than previously appreciated. The miRNAs with high isoform entropy at 5’ end have the greatest significantly function items, most of which are cancer-related functions. Considering many of our input data come from cancer-related research, we suggest that the miRNA cannot only change the expression level but also evolve many isomiR variants to implement the function in a more effective way. Our results agree with previous reports that the isomiRs could play important roles in oncogenesis process [44–46]. The other enriched functions show that the regulation spectrum of the isomiR sequences is related to a very broad diversity of biological processes.

Although isomiRs are highly overlapped with each other and most of them should have similar function, variants in the 5’ end position of miRNAs are expected to alter the canonical seed sequences, which usually complementary binding to the target mRNA genes, thereby considerable versatility in miRNA target selection [16,20–22,47]. Functional enrichment analysis of miRNAs with high *MIH*5 also supports the 5’ end variation should play an important role. An earlier study reported that the biotin-labeled isomiRs can act cooperatively with canonical miRNAs to target functional related but different genes, suggesting biologically relevant and functionally cooperative about these molecules [9]. The existence of multiple 5’ isomiRs could enable miRNA genes greatly expand the targeting potential with the utilization of length heterogeneity and implement the function in a more effective way for some specific networks. In our result, the variation at the 3’ end was not significantly correlated with the MFE and length of pre-miRNA, and the miRNAs with high isoform entropy at the 3’ end can be hardly enriched in function terms. The 3’ isomiRs, which is proved to be equally effective inhibitor to target gene as the canonical miRNA in recent study[48], should affect less than the 5’ isomiRs to the post-transcriptional gene regulation.

Previous position shift based method, like the weighted average size of nucleotide variation (WAZNV), the location of reference miRNA sequence (defined by the most dominant sequence) is the most important factor used to calculate the relative distance of isomiR variants [27,46]. However, switching of the most dominant isomiRs found in several studies makes it hard to define the reference sequence for a specific miRNA [27,43,44], which leads to the difference between the detected reference sequence and the canonical mature miRNA [49]. Also, the KS test based method for isomiR distribution pattern comparison can be only used to examine individual miRNA isoform profile, and then check if the distribution patterns in two different conditions are unique or not[44]. One of the advantages of our strategy is less dependent on the exact location of the most dominant mature miRNA, and the wide range region (canonical mature miRNA ± 4 nt) should include most of the isomiR variants. Therefore, the location of reference miRNA should have limited contribution for the *MIH* value calculation.

## Supporting Information

S1 Fig Plot of the count of aligned sequences and *MIH* for each miRNA. Two lines on x-axis indicate the cut off of 50 and 10000. The solid line shows a lowess smooth of the plot.
(DOC)

S1 Table List of miRNAs and their *MIH* values. (DOC)

S2 Table The KEGG pathway enrichment of the experimentally validated miRNA target genes using the DAVID Bioinformatics Tools. (DOC)

S3 Table The KEGG pathway enrichment of the TargetScan predicted miRNA target genes using the DAVID Bioinformatics Tools. (DOC)
