# Supplementary material for: Entropy-Based Model for MiRNA Isoform Analysis
Source: PLoS One. 2015 Mar 18;10(3):e0118856. doi: 10.1371/journal.pone.0118856 (PMC4364746; doi:10.1371/journal.pone.0118856)
Supplement: S1 Fig — Two lines on x-axis indicate the cut off of 50 and 10000. The solid line shows a lowess smooth of the plot. (DOC) [file pone.0118856.s001.doc]

S1 Fig. Plot of the count of aligned sequences and *MIH* for each miRNA. Two lines on *x*-axis indicate the cut off of 50 and 10000. The solid line shows a lowess smooth of the plot.
