# Supplementary material for: Entropy-Based Model for MiRNA Isoform Analysis
Source: PLoS One. 2015 Mar 18;10(3):e0118856. doi: 10.1371/journal.pone.0118856 (PMC4364746; doi:10.1371/journal.pone.0118856)
Supplement: S1 Table — (DOC) [file pone.0118856.s002.doc]

S1 Table. List of miRNAs and their MIH values.

| MiRNA precursor | Mature miRNA | MFE | MIH | MIH5 | MIH3 | Count |
| --- | --- | --- | --- | --- | --- | --- |
| hsa-mir-1248 | hsa-miR-1248 | -34.5 | 1.0960 | 0.0705 | 0.6694 | 151 |
| hsa-mir-6087 | hsa-miR-6087 | -27.3 | 0.8795 | 0.5181 | 0.4442 | 638 |
| hsa-mir-3202-2 | hsa-miR-3202 | -46.4 | 0.8240 | 0.6409 | 0.7126 | 131 |
| hsa-mir-3202-1 | hsa-miR-3202 | -51.9 | 0.6181 | 0.4881 | 0.5870 | 270 |
| hsa-mir-196a-1 | hsa-miR-196a-5p | -25.8 | 0.6101 | 0.3060 | 0.4633 | 111 |
| hsa-mir-619 | hsa-miR-619-5p | -31.2 | 0.6071 | 0.4550 | 0.6703 | 65 |
| hsa-mir-4532 | hsa-miR-4532 | -28.2 | 0.5854 | 0.3970 | 0.4486 | 58 |
| hsa-mir-4464 | hsa-miR-4464 | -55.4 | 0.5733 | 0.6123 | 0.5852 | 152 |
| hsa-mir-30c-1 | hsa-miR-30c-5p | -35.4 | 0.5403 | 0.3506 | 0.7096 | 326 |
| hsa-mir-30c-2 | hsa-miR-30c-5p | -25.24 | 0.5184 | 0.3571 | 0.7086 | 329 |
| hsa-mir-125b-2 | hsa-miR-125b-5p | -40.6 | 0.5100 | 0.4771 | 0.5424 | 983 |
| hsa-mir-20a | hsa-miR-20a-5p | -31 | 0.5067 | 0.2930 | 0.5653 | 1124 |
| hsa-mir-484 | hsa-miR-484 | -33.8 | 0.4957 | 0.1872 | 0.5461 | 388 |
| hsa-mir-374a | hsa-miR-374a-5p | -35.2 | 0.4926 | 0.2966 | 0.4995 | 405 |
| hsa-mir-451a | hsa-miR-451a | -44.7 | 0.4785 | 0.4990 | 0.5771 | 1442 |
| hsa-mir-139 | hsa-miR-139-5p | -34.9 | 0.4772 | 0.3710 | 0.4568 | 184 |
| hsa-mir-335 | hsa-miR-335-5p | -41.5 | 0.4745 | 0.3412 | 0.5180 | 1185 |
| hsa-mir-100 | hsa-miR-100-5p | -26.8 | 0.4600 | 0.3661 | 0.5689 | 456 |
| hsa-mir-125b-1 | hsa-miR-125b-5p | -43.4 | 0.4545 | 0.4743 | 0.5536 | 1014 |
| hsa-mir-125a | hsa-miR-125a-5p | -48.1 | 0.4525 | 0.3057 | 0.4413 | 1172 |
| hsa-mir-196a-2 | hsa-miR-196a-5p | -51.2 | 0.4475 | 0.3534 | 0.5151 | 127 |
| hsa-mir-1291 | hsa-miR-1291 | -38.7 | 0.4458 | 0.1043 | 0.5971 | 326 |
| hsa-mir-6724 | hsa-miR-6724-5p | -49.8 | 0.4455 | 0.4078 | 0.3361 | 81 |
| hsa-mir-4284 | hsa-miR-4284 | -27.8 | 0.4417 | 0.3736 | 0.4687 | 114 |
| hsa-mir-5091 | hsa-miR-5091 | -29.29 | 0.4414 | 0.2425 | 0.4990 | 54 |
| hsa-mir-18a | hsa-miR-18a-5p | -22 | 0.4406 | 0.3539 | 0.5401 | 422 |
| hsa-mir-4516 | hsa-miR-4516 | -44.6 | 0.4349 | 0.2083 | 0.2266 | 176 |
| hsa-mir-195 | hsa-miR-195-5p | -46.44 | 0.4335 | 0.4122 | 0.5753 | 694 |
| hsa-mir-185 | hsa-miR-185-5p | -53.1 | 0.4231 | 0.1880 | 0.7499 | 942 |
| hsa-mir-4454 | hsa-miR-4454 | -15 | 0.4196 | 0.1794 | 0.2838 | 1121 |
| hsa-mir-374b | hsa-miR-374b-5p | -41.4 | 0.4149 | 0.1916 | 0.5076 | 714 |
| hsa-mir-24-2 | hsa-miR-24-2-5p | -27.3 | 0.4141 | 0.5529 | 0.3440 | 926 |
| hsa-mir-3913-2 | hsa-miR-3913-5p | -73.7 | 0.4129 | 0.3586 | 0.5398 | 84 |
| hsa-mir-3913-1 | hsa-miR-3913-5p | -78.9 | 0.4121 | 0.3587 | 0.5387 | 85 |
| hsa-mir-29c | hsa-miR-29c-3p | -35.4 | 0.4089 | 0.4976 | 0.6001 | 637 |
| hsa-mir-1254-2 | hsa-miR-1254 | -24.6 | 0.4071 | 0.1039 | 0.3906 | 351 |
| hsa-mir-145 | hsa-miR-145-5p | -41.62 | 0.4058 | 0.4295 | 0.4760 | 3081 |
| hsa-mir-151a | hsa-miR-151a-5p | -46.2 | 0.4036 | 0.3142 | 0.6376 | 1309 |
| hsa-mir-16-2 | hsa-miR-16-5p | -30.8 | 0.4029 | 0.3391 | 0.7820 | 1383 |
| hsa-mir-1268b | hsa-miR-1268b | -18.1 | 0.3966 | 0.1075 | 0.3541 | 509 |
| hsa-mir-19b-1 | hsa-miR-19b-3p | -38.42 | 0.3949 | 0.4662 | 0.5394 | 750 |
| hsa-mir-27b | hsa-miR-27b-3p | -50.4 | 0.3917 | 0.5214 | 0.4683 | 780 |
| hsa-mir-19b-2 | hsa-miR-19b-3p | -40.6 | 0.3906 | 0.4281 | 0.6134 | 956 |
| hsa-mir-196b | hsa-miR-196b-5p | -34.3 | 0.3845 | 0.2535 | 0.5026 | 166 |
| hsa-mir-135a-1 | hsa-miR-135a-5p | -44.72 | 0.3785 | 0.4428 | 0.2193 | 124 |
| hsa-mir-29b-2 | hsa-miR-29b-3p | -31.24 | 0.3734 | 0.4529 | 0.5550 | 1555 |
| hsa-mir-3916 | hsa-miR-3916 | -32.2 | 0.3721 | 0.3277 | 0.5309 | 279 |
| hsa-mir-23b | hsa-miR-23b-3p | -36.04 | 0.3714 | 0.4226 | 0.6820 | 1417 |
| hsa-mir-374c | hsa-miR-374c-5p | -37.3 | 0.3713 | 0.3307 | 0.1403 | 58 |
| hsa-mir-16-1 | hsa-miR-16-5p | -37.7 | 0.3707 | 0.2896 | 0.7024 | 1596 |
| hsa-mir-143 | hsa-miR-143-5p | -51.9 | 0.3688 | 0.3291 | 0.4146 | 465 |
| hsa-mir-29b-1 | hsa-miR-29b-3p | -34.02 | 0.3666 | 0.4477 | 0.5461 | 1549 |
| hsa-mir-99a | hsa-miR-99a-5p | -39.6 | 0.3637 | 0.4409 | 0.4089 | 641 |
| hsa-mir-3131 | hsa-miR-3131 | -30.5 | 0.3621 | 0.0765 | 0.3059 | 78 |
| hsa-mir-130a | hsa-miR-130a-3p | -42.6 | 0.3618 | 0.4065 | 0.5957 | 678 |
| hsa-mir-26b | hsa-miR-26b-5p | -40.1 | 0.3564 | 0.3877 | 0.3955 | 1490 |
| hsa-mir-4488 | hsa-miR-4488 | -40.1 | 0.3559 | 0.1867 | 0.2483 | 175 |
| hsa-mir-1268a | hsa-miR-1268a | -25.6 | 0.3515 | 0.1441 | 0.2869 | 472 |
| hsa-mir-455 | hsa-miR-455-5p | -41.6 | 0.3482 | 0.3220 | 0.4783 | 522 |
| hsa-mir-30d | hsa-miR-30d-5p | -28.1 | 0.3470 | 0.1710 | 0.7387 | 1961 |
| hsa-mir-101-1 | hsa-miR-101-3p | -36.2 | 0.3459 | 0.5194 | 0.4604 | 1861 |
| hsa-mir-10b | hsa-miR-10b-5p | -45 | 0.3453 | 0.3378 | 0.4483 | 1827 |
| hsa-mir-24-1 | hsa-miR-24-1-5p | -26.32 | 0.3439 | 0.2468 | 0.3435 | 258 |
| hsa-mir-24-1 | hsa-miR-24-3p | -26.32 | 0.3422 | 0.4174 | 0.5753 | 1237 |
| hsa-mir-143 | hsa-miR-143-3p | -51.9 | 0.3400 | 0.5822 | 0.4266 | 841 |
| hsa-mir-136 | hsa-miR-136-5p | -45.8 | 0.3389 | 0.2738 | 0.3424 | 552 |
| hsa-mir-5571 | hsa-miR-5571-5p | -52.44 | 0.3363 | 0.3390 | 0.5128 | 112 |
| hsa-mir-24-2 | hsa-miR-24-3p | -27.3 | 0.3337 | 0.4181 | 0.5533 | 1213 |
| hsa-mir-1246 | hsa-miR-1246 | -19.21 | 0.3325 | 0.3371 | 0.3256 | 498 |
| hsa-mir-4508 | hsa-miR-4508 | -37.7 | 0.3319 | 0.2963 | 0.2937 | 207 |
| hsa-mir-664b | hsa-miR-664b-5p | -26.1 | 0.3305 | 0.2159 | 0.5427 | 1234 |
| hsa-mir-483 | hsa-miR-483-5p | -44.2 | 0.3293 | 0.2248 | 0.5973 | 268 |
| hsa-mir-200b | hsa-miR-200b-3p | -44.3 | 0.3250 | 0.3285 | 0.6046 | 582 |
| hsa-mir-23a | hsa-miR-23a-3p | -33.2 | 0.3237 | 0.3957 | 0.5765 | 2872 |
| hsa-mir-1255b-1 | hsa-miR-1255b-5p | -17.2 | 0.3211 | 0.0475 | 0.2944 | 551 |
| hsa-mir-99b | hsa-miR-99b-5p | -30 | 0.3184 | 0.1840 | 0.6199 | 827 |
| hsa-mir-27a | hsa-miR-27a-3p | -37.5 | 0.3171 | 0.4234 | 0.5052 | 478 |
| hsa-mir-21 | hsa-miR-21-5p | -35.8 | 0.3146 | 0.4275 | 0.3350 | 8884 |
| hsa-mir-15a | hsa-miR-15a-5p | -30.74 | 0.3120 | 0.2452 | 0.4825 | 1011 |
| hsa-mir-15b | hsa-miR-15b-5p | -32.7 | 0.3087 | 0.3424 | 0.4017 | 1509 |
| hsa-let-7c | hsa-let-7c-5p | -33.5 | 0.3075 | 0.4830 | 0.4619 | 1789 |
| hsa-mir-101-1 | hsa-miR-101-5p | -36.2 | 0.3064 | 0.1731 | 0.4553 | 322 |
| hsa-mir-3147 | hsa-miR-3147 | -36.8 | 0.3049 | 0.0979 | 0.2632 | 62 |
| hsa-mir-486 | hsa-miR-486-5p | -48.5 | 0.3039 | 0.2111 | 0.6022 | 459 |
| hsa-mir-17 | hsa-miR-17-3p | -34.3 | 0.3030 | 0.3156 | 0.5886 | 980 |
| hsa-mir-135a-2 | hsa-miR-135a-5p | -35.1 | 0.3028 | 0.4428 | 0.2193 | 124 |
| hsa-mir-144 | hsa-miR-144-3p | -44.4 | 0.3028 | 0.4304 | 0.4614 | 669 |
| hsa-mir-3143 | hsa-miR-3143 | -21.1 | 0.2998 | 0.1022 | 0.2254 | 213 |
| hsa-mir-550a-1 | hsa-miR-550a-5p | -46 | 0.2992 | 0.3440 | 0.2752 | 65 |
| hsa-mir-17 | hsa-miR-17-5p | -34.3 | 0.2990 | 0.1461 | 0.4632 | 3146 |
| hsa-mir-550a-2 | hsa-miR-550a-5p | -53.1 | 0.2977 | 0.3448 | 0.2684 | 64 |
| hsa-mir-548ay | hsa-miR-548ay-5p | -50 | 0.2974 | 0.2975 | 0.4078 | 140 |
| hsa-mir-126 | hsa-miR-126-3p | -40.2 | 0.2968 | 0.3879 | 0.4610 | 1124 |
| hsa-mir-218-2 | hsa-miR-218-5p | -46.3 | 0.2951 | 0.4187 | 0.3673 | 505 |
| hsa-mir-106b | hsa-miR-106b-5p | -44.9 | 0.2942 | 0.1619 | 0.6399 | 808 |
| hsa-mir-199a-2 | hsa-miR-199a-5p | -43.2 | 0.2931 | 0.2179 | 0.4264 | 859 |
| hsa-mir-1292 | hsa-miR-1292-5p | -34.6 | 0.2924 | 0.3398 | 0.4074 | 178 |
| hsa-mir-199a-1 | hsa-miR-199a-5p | -29.2 | 0.2917 | 0.2610 | 0.4531 | 980 |
| hsa-mir-127 | hsa-miR-127-5p | -41.1 | 0.2916 | 0.2796 | 0.3683 | 257 |
| hsa-mir-194-1 | hsa-miR-194-5p | -38.3 | 0.2897 | 0.2751 | 0.3477 | 83 |
| hsa-mir-29a | hsa-miR-29a-3p | -24.9 | 0.2895 | 0.4194 | 0.4742 | 5810 |
| hsa-mir-548d-1 | hsa-miR-548d-5p | -48.2 | 0.2890 | 0.2371 | 0.4241 | 185 |
| hsa-mir-218-1 | hsa-miR-218-5p | -42.9 | 0.2881 | 0.4256 | 0.3697 | 509 |
| hsa-mir-548d-2 | hsa-miR-548d-5p | -56.9 | 0.2876 | 0.2379 | 0.4197 | 184 |
| hsa-mir-205 | hsa-miR-205-5p | -49.02 | 0.2846 | 0.2760 | 0.3732 | 2793 |
| hsa-mir-424 | hsa-miR-424-5p | -42.3 | 0.2832 | 0.3051 | 0.3123 | 5057 |
| hsa-mir-199b | hsa-miR-199b-5p | -42.9 | 0.2802 | 0.1854 | 0.4029 | 373 |
| hsa-mir-2110 | hsa-miR-2110 | -34.1 | 0.2787 | 0.0346 | 0.4185 | 733 |
| hsa-mir-126 | hsa-miR-126-5p | -40.2 | 0.2785 | 0.3145 | 0.4026 | 1112 |
| hsa-mir-4791 | hsa-miR-4791 | -26 | 0.2784 | 0.1093 | 0.3547 | 87 |
| hsa-mir-338 | hsa-miR-338-3p | -26.22 | 0.2784 | 0.3108 | 0.4643 | 494 |
| hsa-mir-26a-2 | hsa-miR-26a-5p | -42.24 | 0.2771 | 0.3249 | 0.3513 | 1996 |
| hsa-mir-4443 | hsa-miR-4443 | -25.1 | 0.2769 | 0.2488 | 0.2434 | 841 |
| hsa-mir-362 | hsa-miR-362-5p | -31.7 | 0.2760 | 0.0481 | 0.5668 | 595 |
| hsa-mir-19a | hsa-miR-19a-3p | -39.1 | 0.2747 | 0.3242 | 0.4395 | 1002 |
| hsa-mir-365a | hsa-miR-365a-3p | -35.4 | 0.2731 | 0.2386 | 0.4871 | 1359 |
| hsa-mir-1247 | hsa-miR-1247-5p | -57.7 | 0.2728 | 0.0368 | 0.4687 | 158 |
| hsa-mir-133a-2 | hsa-miR-133a-5p | -47.5 | 0.2726 | 0.2017 | 0.4381 | 84 |
| hsa-mir-365b | hsa-miR-365b-3p | -40.1 | 0.2716 | 0.2355 | 0.4889 | 1357 |
| hsa-mir-361 | hsa-miR-361-5p | -32.3 | 0.2712 | 0.2395 | 0.4449 | 581 |
| hsa-mir-652 | hsa-miR-652-5p | -55.2 | 0.2705 | 0.0855 | 0.3599 | 450 |
| hsa-mir-30b | hsa-miR-30b-5p | -37.6 | 0.2704 | 0.1855 | 0.4182 | 681 |
| hsa-mir-1275 | hsa-miR-1275 | -26.43 | 0.2684 | 0.2054 | 0.5401 | 383 |
| hsa-mir-193b | hsa-miR-193b-3p | -43.1 | 0.2666 | 0.1514 | 0.6463 | 635 |
| hsa-mir-574 | hsa-miR-574-5p | -61.1 | 0.2660 | 0.0873 | 0.4622 | 1401 |
| hsa-mir-450a-2 | hsa-miR-450a-5p | -35.4 | 0.2657 | 0.1119 | 0.4118 | 660 |
| hsa-mir-152 | hsa-miR-152-3p | -48.2 | 0.2657 | 0.2260 | 0.5825 | 1147 |
| hsa-mir-582 | hsa-miR-582-5p | -44.04 | 0.2639 | 0.2384 | 0.3315 | 557 |
| hsa-mir-200a | hsa-miR-200a-3p | -47.5 | 0.2632 | 0.2706 | 0.4363 | 305 |
| hsa-mir-223 | hsa-miR-223-3p | -47.9 | 0.2624 | 0.2988 | 0.4670 | 3039 |
| hsa-mir-34b | hsa-miR-34b-5p | -33.8 | 0.2620 | 0.1454 | 0.3615 | 292 |
| hsa-mir-5090 | hsa-miR-5090 | -35.3 | 0.2618 | 0.1535 | 0.3702 | 152 |
| hsa-mir-200c | hsa-miR-200c-3p | -31.3 | 0.2602 | 0.3587 | 0.3826 | 1349 |
| hsa-mir-4521 | hsa-miR-4521 | -17.2 | 0.2602 | 0.0718 | 0.2462 | 2093 |
| hsa-mir-150 | hsa-miR-150-5p | -57.5 | 0.2594 | 0.2055 | 0.3600 | 1464 |
| hsa-mir-28 | hsa-miR-28-5p | -50.6 | 0.2594 | 0.2336 | 0.4338 | 899 |
| hsa-mir-26a-1 | hsa-miR-26a-5p | -37.3 | 0.2585 | 0.2930 | 0.3567 | 1802 |
| hsa-mir-10a | hsa-miR-10a-5p | -32.39 | 0.2574 | 0.2119 | 0.4431 | 3779 |
| hsa-mir-378g | hsa-miR-378g | -27.4 | 0.2565 | 0.0944 | 0.1377 | 137 |
| hsa-mir-450a-1 | hsa-miR-450a-5p | -35.4 | 0.2553 | 0.0834 | 0.4147 | 646 |
| hsa-mir-133a-1 | hsa-miR-133a-5p | -40.5 | 0.2553 | 0.1810 | 0.4400 | 83 |
| hsa-mir-3613 | hsa-miR-3613-5p | -27.6 | 0.2552 | 0.1515 | 0.4216 | 130 |
| hsa-mir-675 | hsa-miR-675-5p | -48.3 | 0.2552 | 0.0399 | 0.4387 | 460 |
| hsa-mir-30a | hsa-miR-30a-5p | -37.3 | 0.2548 | 0.1694 | 0.5367 | 1982 |
| hsa-mir-502 | hsa-miR-502-5p | -59 | 0.2540 | 0.1281 | 0.5134 | 124 |
| hsa-let-7e | hsa-let-7e-5p | -37.8 | 0.2537 | 0.3059 | 0.4552 | 1747 |
| hsa-mir-4667 | hsa-miR-4667-5p | -31 | 0.2528 | 0.2861 | 0.4887 | 62 |
| hsa-mir-3691 | hsa-miR-3691-5p | -62.3 | 0.2525 | 0.3238 | 0.3811 | 85 |
| hsa-mir-342 | hsa-miR-342-3p | -47.7 | 0.2519 | 0.2681 | 0.5181 | 2057 |
| hsa-mir-6503 | hsa-miR-6503-3p | -62.3 | 0.2516 | 0.3354 | 0.4753 | 252 |
| hsa-mir-214 | hsa-miR-214-3p | -67.62 | 0.2509 | 0.3360 | 0.3775 | 426 |
| hsa-mir-145 | hsa-miR-145-3p | -41.62 | 0.2482 | 0.3014 | 0.4597 | 1011 |
| hsa-mir-133a-1 | hsa-miR-133a-3p | -40.5 | 0.2472 | 0.2144 | 0.5164 | 455 |
| hsa-mir-133a-2 | hsa-miR-133a-3p | -47.5 | 0.2472 | 0.2144 | 0.5164 | 455 |
| hsa-mir-876 | hsa-miR-876-3p | -42.7 | 0.2469 | 0.3722 | 0.3034 | 89 |
| hsa-mir-4326 | hsa-miR-4326 | -24.7 | 0.2437 | 0.1257 | 0.3072 | 61 |
| hsa-mir-376a-1 | hsa-miR-376a-5p | -18.2 | 0.2430 | 0.2039 | 0.3781 | 56 |
| hsa-mir-579 | hsa-miR-579-5p | -49.3 | 0.2415 | 0.1288 | 0.4885 | 84 |
| hsa-mir-144 | hsa-miR-144-5p | -44.4 | 0.2404 | 0.1567 | 0.4194 | 78 |
| hsa-mir-182 | hsa-miR-182-5p | -47.14 | 0.2389 | 0.0160 | 0.6295 | 1730 |
| hsa-mir-34a | hsa-miR-34a-5p | -50.7 | 0.2361 | 0.2117 | 0.4210 | 544 |
| hsa-mir-455 | hsa-miR-455-3p | -41.6 | 0.2347 | 0.2838 | 0.4408 | 3098 |
| hsa-mir-4525 | hsa-miR-4525 | -48.4 | 0.2346 | 0.1280 | 0.2370 | 128 |
| hsa-mir-218-1 | hsa-miR-218-1-3p | -42.9 | 0.2344 | 0.3028 | 0.2868 | 52 |
| hsa-mir-340 | hsa-miR-340-5p | -36 | 0.2341 | 0.1842 | 0.4813 | 322 |
| hsa-mir-500a | hsa-miR-500a-5p | -38.7 | 0.2340 | 0.0623 | 0.5113 | 238 |
| hsa-mir-142 | hsa-miR-142-3p | -44.7 | 0.2339 | 0.2666 | 0.4876 | 5172 |
| hsa-mir-550a-3 | hsa-miR-550a-3-5p | -43.9 | 0.2318 | 0.3088 | 0.3395 | 57 |
| hsa-mir-873 | hsa-miR-873-5p | -31.1 | 0.2300 | 0.1214 | 0.4061 | 248 |
| hsa-mir-190a | hsa-miR-190a-5p | -32.5 | 0.2291 | 0.1077 | 0.4050 | 243 |
| hsa-mir-22 | hsa-miR-22-3p | -39.8 | 0.2289 | 0.1467 | 0.5283 | 1918 |
| hsa-mir-4750 | hsa-miR-4750-5p | -29.2 | 0.2286 | 0.1684 | 0.2889 | 59 |
| hsa-mir-449a | hsa-miR-449a | -39 | 0.2282 | 0.0556 | 0.5089 | 96 |
| hsa-mir-3687 | hsa-miR-3687 | -33.7 | 0.2278 | 0.3505 | 0.3596 | 214 |
| hsa-mir-186 | hsa-miR-186-3p | -41.24 | 0.2274 | 0.1950 | 0.5015 | 134 |
| hsa-mir-28 | hsa-miR-28-3p | -50.6 | 0.2269 | 0.2218 | 0.4473 | 1106 |
| hsa-mir-497 | hsa-miR-497-5p | -55.5 | 0.2258 | 0.2729 | 0.3304 | 863 |
| hsa-mir-574 | hsa-miR-574-3p | -61.1 | 0.2253 | 0.1622 | 0.5093 | 482 |
| hsa-mir-4417 | hsa-miR-4417 | -26.7 | 0.2245 | 0.2280 | 0.2461 | 52 |
| hsa-mir-3607 | hsa-miR-3607-5p | -19.2 | 0.2243 | 0.1198 | 0.2580 | 116 |
| hsa-mir-9-2 | hsa-miR-9-5p | -40.1 | 0.2233 | 0.1363 | 0.3218 | 1312 |
| hsa-mir-542 | hsa-miR-542-5p | -35.9 | 0.2228 | 0.0715 | 0.4235 | 258 |
| hsa-mir-200a | hsa-miR-200a-5p | -47.5 | 0.2212 | 0.1207 | 0.4366 | 150 |
| hsa-mir-409 | hsa-miR-409-5p | -38.3 | 0.2200 | 0.0338 | 0.3568 | 241 |
| hsa-mir-151a | hsa-miR-151a-3p | -46.2 | 0.2183 | 0.3284 | 0.3648 | 2589 |
| hsa-mir-19b-1 | hsa-miR-19b-1-5p | -38.42 | 0.2181 | 0.0555 | 0.4133 | 72 |
| hsa-mir-3690-1 | hsa-miR-3690 | -32.8 | 0.2176 | 0.0033 | 0.4077 | 327 |
| hsa-mir-887 | hsa-miR-887-5p | -40.9 | 0.2176 | 0.1860 | 0.3732 | 74 |
| hsa-mir-299 | hsa-miR-299-5p | -40.5 | 0.2165 | 0.1113 | 0.2996 | 193 |
| hsa-mir-106a | hsa-miR-106a-5p | -34.7 | 0.2153 | 0.1653 | 0.2310 | 1507 |
| hsa-mir-942 | hsa-miR-942-5p | -56.44 | 0.2151 | 0.2222 | 0.3934 | 399 |
| hsa-mir-25 | hsa-miR-25-3p | -37.8 | 0.2138 | 0.1024 | 0.5949 | 7375 |
| hsa-mir-214 | hsa-miR-214-5p | -67.62 | 0.2131 | 0.1754 | 0.4380 | 79 |
| hsa-mir-103a-2 | hsa-miR-103a-2-5p | -28.1 | 0.2127 | 0.0214 | 0.4437 | 132 |
| hsa-mir-130b | hsa-miR-130b-3p | -36.4 | 0.2112 | 0.2059 | 0.4530 | 1028 |
| hsa-let-7i | hsa-let-7i-5p | -39.6 | 0.2111 | 0.3743 | 0.5356 | 1337 |
| hsa-mir-671 | hsa-miR-671-5p | -68.1 | 0.2109 | 0.0407 | 0.4258 | 212 |
| hsa-mir-339 | hsa-miR-339-5p | -49.2 | 0.2091 | 0.0881 | 0.3473 | 910 |
| hsa-mir-532 | hsa-miR-532-5p | -25.72 | 0.2090 | 0.1321 | 0.3732 | 1271 |
| hsa-mir-501 | hsa-miR-501-5p | -43.1 | 0.2087 | 0.0846 | 0.4205 | 227 |
| hsa-mir-654 | hsa-miR-654-3p | -29.6 | 0.2073 | 0.2326 | 0.4420 | 94 |
| hsa-mir-452 | hsa-miR-452-5p | -40.9 | 0.2066 | 0.3121 | 0.3618 | 1603 |
| hsa-mir-30e | hsa-miR-30e-3p | -53 | 0.2060 | 0.2475 | 0.4043 | 2113 |
| hsa-mir-548w | hsa-miR-548w | -38 | 0.2058 | 0.0971 | 0.2777 | 58 |
| hsa-mir-29c | hsa-miR-29c-5p | -35.4 | 0.2058 | 0.2431 | 0.2797 | 1416 |
| hsa-mir-4485 | hsa-miR-4485 | -16.9 | 0.2057 | 0.4680 | 0.3846 | 995 |
| hsa-mir-660 | hsa-miR-660-5p | -33.1 | 0.2053 | 0.1807 | 0.2808 | 821 |
| hsa-mir-9-3 | hsa-miR-9-5p | -41.1 | 0.2053 | 0.1354 | 0.3245 | 1318 |
| hsa-mir-29b-1 | hsa-miR-29b-1-5p | -34.02 | 0.2051 | 0.0522 | 0.4400 | 235 |
| hsa-mir-9-1 | hsa-miR-9-5p | -42 | 0.2039 | 0.1358 | 0.3201 | 1311 |
| hsa-mir-876 | hsa-miR-876-5p | -42.7 | 0.2039 | 0.1255 | 0.4183 | 272 |
| hsa-mir-3129 | hsa-miR-3129-5p | -49.1 | 0.2038 | 0.2174 | 0.3261 | 78 |
| hsa-mir-204 | hsa-miR-204-3p | -42.26 | 0.2035 | 0.1206 | 0.4628 | 256 |
| hsa-mir-23a | hsa-miR-23a-5p | -33.2 | 0.2035 | 0.1536 | 0.2831 | 244 |
| hsa-mir-30e | hsa-miR-30e-5p | -53 | 0.2029 | 0.1466 | 0.4326 | 3106 |
| hsa-mir-4429 | hsa-miR-4429 | -25.2 | 0.2029 | 0.1191 | 0.1965 | 93 |
| hsa-mir-339 | hsa-miR-339-3p | -49.2 | 0.2025 | 0.2446 | 0.4167 | 1941 |
| hsa-mir-376c | hsa-miR-376c-3p | -24.02 | 0.2023 | 0.1862 | 0.4402 | 580 |
| hsa-mir-193a | hsa-miR-193a-3p | -50.8 | 0.2022 | 0.1873 | 0.3430 | 1709 |
| hsa-mir-3200 | hsa-miR-3200-5p | -34.5 | 0.2006 | 0.1381 | 0.4516 | 69 |
| hsa-mir-193a | hsa-miR-193a-5p | -50.8 | 0.2005 | 0.1403 | 0.2752 | 1013 |
| hsa-mir-190b | hsa-miR-190b | -32.6 | 0.2003 | 0.1513 | 0.1530 | 79 |
| hsa-mir-449b | hsa-miR-449b-5p | -35.6 | 0.2002 | 0.0610 | 0.4838 | 75 |
| hsa-mir-93 | hsa-miR-93-5p | -44.8 | 0.1999 | 0.0778 | 0.3770 | 2892 |
| hsa-mir-149 | hsa-miR-149-5p | -55.8 | 0.1999 | 0.0349 | 0.4117 | 450 |
| hsa-mir-132 | hsa-miR-132-5p | -48.4 | 0.1979 | 0.1791 | 0.2803 | 236 |
| hsa-mir-505 | hsa-miR-505-3p | -44.9 | 0.1976 | 0.2087 | 0.4431 | 1246 |
| hsa-mir-548l | hsa-miR-548l | -41.6 | 0.1965 | 0.0000 | 0.4804 | 233 |
| hsa-mir-331 | hsa-miR-331-5p | -43.52 | 0.1962 | 0.1479 | 0.2904 | 308 |
| hsa-mir-141 | hsa-miR-141-3p | -48.9 | 0.1958 | 0.1627 | 0.4255 | 1745 |
| hsa-mir-1-1 | hsa-miR-1 | -30 | 0.1956 | 0.4412 | 0.5393 | 775 |
| hsa-mir-548c | hsa-miR-548c-5p | -46.9 | 0.1952 | 0.1259 | 0.3419 | 94 |
| hsa-mir-590 | hsa-miR-590-5p | -31.8 | 0.1944 | 0.2469 | 0.2767 | 393 |
| hsa-mir-95 | hsa-miR-95-3p | -37.5 | 0.1942 | 0.2328 | 0.3889 | 270 |
| hsa-mir-301a | hsa-miR-301a-3p | -32.8 | 0.1940 | 0.0850 | 0.5118 | 704 |
| hsa-mir-183 | hsa-miR-183-5p | -41.9 | 0.1935 | 0.1321 | 0.3771 | 786 |
| hsa-mir-4497 | hsa-miR-4497 | -59.7 | 0.1901 | 0.1261 | 0.0851 | 167 |
| hsa-mir-641 | hsa-miR-641 | -61.8 | 0.1896 | 0.1107 | 0.3518 | 460 |
| hsa-mir-29a | hsa-miR-29a-5p | -24.9 | 0.1895 | 0.0774 | 0.4071 | 1950 |
| hsa-mir-205 | hsa-miR-205-3p | -49.02 | 0.1882 | 0.1677 | 0.4186 | 201 |
| hsa-mir-18b | hsa-miR-18b-5p | -19.8 | 0.1867 | 0.0237 | 0.4380 | 495 |
| hsa-mir-2116 | hsa-miR-2116-3p | -42.6 | 0.1864 | 0.1782 | 0.4224 | 94 |
| hsa-mir-500b | hsa-miR-500b-3p | -40.8 | 0.1860 | 0.2744 | 0.3044 | 59 |
| hsa-mir-101-2 | hsa-miR-101-3p | -31.4 | 0.1860 | 0.5219 | 0.4954 | 1903 |
| hsa-mir-378d-2 | hsa-miR-378d | -30.2 | 0.1856 | 0.1058 | 0.4922 | 245 |
| hsa-mir-766 | hsa-miR-766-5p | -50.8 | 0.1856 | 0.0297 | 0.4230 | 4291 |
| hsa-mir-92a-2 | hsa-miR-92a-3p | -30.2 | 0.1842 | 0.1749 | 0.3936 | 4543 |
| hsa-mir-30a | hsa-miR-30a-3p | -37.3 | 0.1833 | 0.1645 | 0.4128 | 2400 |
| hsa-mir-1270-2 | hsa-miR-1270 | -33.5 | 0.1817 | 0.1024 | 0.3014 | 875 |
| hsa-mir-29b-2 | hsa-miR-29b-2-5p | -31.24 | 0.1816 | 0.1219 | 0.3984 | 360 |
| hsa-mir-99a | hsa-miR-99a-3p | -39.6 | 0.1813 | 0.0868 | 0.4720 | 288 |
| hsa-mir-1254-1 | hsa-miR-1254 | -39.5 | 0.1802 | 0.1004 | 0.3970 | 351 |
| hsa-mir-505 | hsa-miR-505-5p | -44.9 | 0.1797 | 0.0865 | 0.3116 | 1356 |
| hsa-mir-10a | hsa-miR-10a-3p | -32.39 | 0.1790 | 0.2348 | 0.3456 | 168 |
| hsa-mir-3605 | hsa-miR-3605-5p | -30.56 | 0.1787 | 0.2258 | 0.3327 | 322 |
| hsa-mir-125b-2 | hsa-miR-125b-2-3p | -40.6 | 0.1784 | 0.2449 | 0.3369 | 322 |
| hsa-mir-1228 | hsa-miR-1228-3p | -46 | 0.1782 | 0.1864 | 0.3681 | 52 |
| hsa-mir-93 | hsa-miR-93-3p | -44.8 | 0.1781 | 0.1808 | 0.3784 | 353 |
| hsa-mir-210 | hsa-miR-210-3p | -60.7 | 0.1780 | 0.0964 | 0.4660 | 1518 |
| hsa-mir-425 | hsa-miR-425-5p | -36.3 | 0.1774 | 0.1003 | 0.3978 | 5557 |
| hsa-mir-5009 | hsa-miR-5009-5p | -31.52 | 0.1772 | 0.1786 | 0.2938 | 55 |
| hsa-mir-215 | hsa-miR-215-5p | -33.4 | 0.1769 | 0.0876 | 0.3683 | 363 |
| hsa-mir-548o-2 | hsa-miR-548o-5p | -39.3 | 0.1766 | 0.1177 | 0.3438 | 93 |
| hsa-mir-494 | hsa-miR-494-3p | -34.1 | 0.1761 | 0.1276 | 0.4181 | 152 |
| hsa-mir-5001 | hsa-miR-5001-5p | -53.4 | 0.1754 | 0.0151 | 0.4254 | 123 |
| hsa-mir-1270-1 | hsa-miR-1270 | -33.5 | 0.1748 | 0.0901 | 0.2983 | 827 |
| hsa-mir-27b | hsa-miR-27b-5p | -50.4 | 0.1747 | 0.1352 | 0.3954 | 3406 |
| hsa-mir-4517 | hsa-miR-4517 | -22.2 | 0.1740 | 0.0715 | 0.1605 | 61 |
| hsa-mir-4647 | hsa-miR-4647 | -41.3 | 0.1739 | 0.0341 | 0.3137 | 222 |
| hsa-mir-1-2 | hsa-miR-1 | -35.64 | 0.1738 | 0.4460 | 0.5389 | 756 |
| hsa-mir-3140 | hsa-miR-3140-3p | -43 | 0.1735 | 0.3139 | 0.2644 | 166 |
| hsa-mir-500a | hsa-miR-500a-3p | -38.7 | 0.1725 | 0.1107 | 0.4578 | 2403 |
| hsa-mir-127 | hsa-miR-127-3p | -41.1 | 0.1709 | 0.1515 | 0.3731 | 450 |
| hsa-mir-96 | hsa-miR-96-5p | -34.4 | 0.1702 | 0.0558 | 0.3986 | 606 |
| hsa-mir-3664 | hsa-miR-3664-3p | -53.2 | 0.1701 | 0.3298 | 0.2374 | 113 |
| hsa-mir-199a-2 | hsa-miR-199a-3p | -43.2 | 0.1698 | 0.2057 | 0.3231 | 3298 |
| hsa-mir-548am | hsa-miR-548am-5p | -48.2 | 0.1697 | 0.0993 | 0.3054 | 87 |
| hsa-mir-381 | hsa-miR-381-3p | -35 | 0.1690 | 0.0943 | 0.3854 | 280 |
| hsa-mir-199b | hsa-miR-199b-3p | -42.9 | 0.1690 | 0.2052 | 0.3205 | 3293 |
| hsa-mir-199a-1 | hsa-miR-199a-3p | -29.2 | 0.1688 | 0.2053 | 0.3200 | 3292 |
| hsa-mir-135b | hsa-miR-135b-5p | -47.2 | 0.1687 | 0.2010 | 0.1572 | 443 |
| hsa-mir-10b | hsa-miR-10b-3p | -45 | 0.1687 | 0.2341 | 0.3084 | 922 |
| hsa-mir-497 | hsa-miR-497-3p | -55.5 | 0.1685 | 0.2024 | 0.3400 | 151 |
| hsa-mir-664a | hsa-miR-664a-5p | -24.4 | 0.1683 | 0.1181 | 0.2916 | 8868 |
| hsa-mir-503 | hsa-miR-503-5p | -42.1 | 0.1677 | 0.0190 | 0.4281 | 3965 |
| hsa-mir-138-1 | hsa-miR-138-5p | -58.8 | 0.1676 | 0.0395 | 0.4093 | 4706 |
| hsa-mir-6503 | hsa-miR-6503-5p | -62.3 | 0.1672 | 0.1067 | 0.2794 | 264 |
| hsa-mir-324 | hsa-miR-324-3p | -38.6 | 0.1672 | 0.2360 | 0.2784 | 587 |
| hsa-mir-33b | hsa-miR-33b-5p | -59.1 | 0.1664 | 0.0467 | 0.4340 | 203 |
| hsa-mir-708 | hsa-miR-708-5p | -44.1 | 0.1662 | 0.0643 | 0.4087 | 747 |
| hsa-mir-6515 | hsa-miR-6515-5p | -21.8 | 0.1647 | 0.0821 | 0.3103 | 84 |
| hsa-mir-1296 | hsa-miR-1296-5p | -50.2 | 0.1645 | 0.0660 | 0.3709 | 223 |
| hsa-mir-4710 | hsa-miR-4710 | -25.4 | 0.1643 | 0.1077 | 0.2027 | 66 |
| hsa-mir-4286 | hsa-miR-4286 | -27 | 0.1641 | 0.0545 | 0.2738 | 1332 |
| hsa-mir-105-1 | hsa-miR-105-5p | -43.1 | 0.1640 | 0.2697 | 0.1307 | 95 |
| hsa-mir-105-2 | hsa-miR-105-5p | -41.3 | 0.1640 | 0.2697 | 0.1307 | 95 |
| hsa-mir-1260b | hsa-miR-1260b | -42.9 | 0.1636 | 0.0529 | 0.2560 | 454 |
| hsa-mir-296 | hsa-miR-296-3p | -41.4 | 0.1633 | 0.1179 | 0.3953 | 251 |
| hsa-mir-20a | hsa-miR-20a-3p | -31 | 0.1630 | 0.1511 | 0.3635 | 655 |
| hsa-mir-26a-2 | hsa-miR-26a-2-3p | -42.24 | 0.1629 | 0.2474 | 0.2885 | 262 |
| hsa-mir-328 | hsa-miR-328-3p | -46.3 | 0.1627 | 0.0541 | 0.3967 | 174 |
| hsa-mir-222 | hsa-miR-222-5p | -54.1 | 0.1624 | 0.2352 | 0.1977 | 280 |
| hsa-mir-130b | hsa-miR-130b-5p | -36.4 | 0.1609 | 0.0377 | 0.3931 | 186 |
| hsa-mir-15a | hsa-miR-15a-3p | -30.74 | 0.1606 | 0.1394 | 0.3745 | 135 |
| hsa-mir-3065 | hsa-miR-3065-5p | -35.1 | 0.1606 | 0.0413 | 0.4047 | 59 |
| hsa-mir-3144 | hsa-miR-3144-5p | -37.1 | 0.1595 | 0.1111 | 0.2611 | 141 |
| hsa-mir-548au | hsa-miR-548au-5p | -16.9 | 0.1593 | 0.0856 | 0.2684 | 65 |
| hsa-mir-338 | hsa-miR-338-5p | -26.22 | 0.1583 | 0.1471 | 0.2261 | 140 |
| hsa-mir-212 | hsa-miR-212-3p | -50.8 | 0.1579 | 0.1512 | 0.3417 | 246 |
| hsa-mir-5699 | hsa-miR-5699-5p | -50.3 | 0.1577 | 0.1105 | 0.4579 | 53 |
| hsa-mir-186 | hsa-miR-186-5p | -41.24 | 0.1574 | 0.0744 | 0.3485 | 6806 |
| hsa-mir-324 | hsa-miR-324-5p | -38.6 | 0.1573 | 0.0751 | 0.2626 | 1236 |
| hsa-mir-210 | hsa-miR-210-5p | -60.7 | 0.1561 | 0.0000 | 0.3868 | 54 |
| hsa-mir-31 | hsa-miR-31-5p | -36 | 0.1558 | 0.0349 | 0.3538 | 4759 |
| hsa-mir-365a | hsa-miR-365a-5p | -35.4 | 0.1551 | 0.0768 | 0.3527 | 2894 |
| hsa-mir-3192 | hsa-miR-3192-5p | -31.9 | 0.1550 | 0.0337 | 0.3623 | 296 |
| hsa-mir-1973 | hsa-miR-1973 | -10.8 | 0.1548 | 0.2174 | 0.3457 | 223 |
| hsa-mir-1262 | hsa-miR-1262 | -23.31 | 0.1547 | 0.1871 | 0.1702 | 283 |
| hsa-mir-1304 | hsa-miR-1304-5p | -73.6 | 0.1545 | 0.1213 | 0.1961 | 521 |
| hsa-mir-187 | hsa-miR-187-3p | -47.91 | 0.1545 | 0.0737 | 0.4051 | 186 |
| hsa-mir-31 | hsa-miR-31-3p | -36 | 0.1543 | 0.1137 | 0.3788 | 648 |
| hsa-mir-202 | hsa-miR-202-3p | -58.8 | 0.1542 | 0.1385 | 0.3220 | 91 |
| hsa-mir-874 | hsa-miR-874-3p | -37.2 | 0.1537 | 0.0395 | 0.4414 | 315 |
| hsa-mir-539 | hsa-miR-539-3p | -29.2 | 0.1534 | 0.2727 | 0.2388 | 1813 |
| hsa-mir-425 | hsa-miR-425-3p | -36.3 | 0.1530 | 0.1448 | 0.3502 | 2749 |
| hsa-mir-545 | hsa-miR-545-5p | -37.52 | 0.1527 | 0.1233 | 0.2495 | 74 |
| hsa-mir-1271 | hsa-miR-1271-3p | -50.2 | 0.1526 | 0.0557 | 0.4530 | 91 |
| hsa-mir-590 | hsa-miR-590-3p | -31.8 | 0.1525 | 0.1996 | 0.2626 | 296 |
| hsa-mir-1269b | hsa-miR-1269b | -28.5 | 0.1522 | 0.0000 | 0.2504 | 87 |
| hsa-mir-197 | hsa-miR-197-5p | -42.4 | 0.1520 | 0.0401 | 0.3359 | 369 |
| hsa-mir-194-2 | hsa-miR-194-5p | -51.3 | 0.1518 | 0.1354 | 0.1612 | 499 |
| hsa-mir-20b | hsa-miR-20b-3p | -29.8 | 0.1512 | 0.0773 | 0.4256 | 2436 |
| hsa-mir-34b | hsa-miR-34b-3p | -33.8 | 0.1508 | 0.1725 | 0.3135 | 309 |
| hsa-mir-615 | hsa-miR-615-3p | -58.8 | 0.1504 | 0.0197 | 0.3939 | 278 |
| hsa-mir-874 | hsa-miR-874-5p | -37.2 | 0.1501 | 0.0793 | 0.2876 | 57 |
| hsa-mir-450a-1 | hsa-miR-450a-1-3p | -35.4 | 0.1496 | 0.1542 | 0.2842 | 65 |
| hsa-mir-708 | hsa-miR-708-3p | -44.1 | 0.1493 | 0.1525 | 0.3452 | 153 |
| hsa-mir-320d-2 | hsa-miR-320d | -23.1 | 0.1491 | 0.2386 | 0.3744 | 580 |
| hsa-mir-320d-1 | hsa-miR-320d | -20.1 | 0.1487 | 0.2366 | 0.3747 | 579 |
| hsa-mir-1277 | hsa-miR-1277-5p | -34.6 | 0.1486 | 0.0615 | 0.2166 | 109 |
| hsa-mir-191 | hsa-miR-191-3p | -49 | 0.1484 | 0.1265 | 0.3576 | 186 |
| hsa-mir-483 | hsa-miR-483-3p | -44.2 | 0.1481 | 0.1232 | 0.3489 | 202 |
| hsa-mir-502 | hsa-miR-502-3p | -59 | 0.1478 | 0.1049 | 0.3831 | 1928 |
| hsa-mir-629 | hsa-miR-629-5p | -74.8 | 0.1477 | 0.0315 | 0.3952 | 2063 |
| hsa-mir-32 | hsa-miR-32-5p | -31.9 | 0.1474 | 0.0630 | 0.3548 | 885 |
| hsa-mir-382 | hsa-miR-382-5p | -28.5 | 0.1474 | 0.1395 | 0.2162 | 764 |
| hsa-mir-3173 | hsa-miR-3173-3p | -47.6 | 0.1463 | 0.1702 | 0.3175 | 57 |
| hsa-mir-409 | hsa-miR-409-3p | -38.3 | 0.1462 | 0.1499 | 0.3260 | 871 |
| hsa-let-7g | hsa-let-7g-5p | -40.5 | 0.1461 | 0.2548 | 0.3976 | 4546 |
| hsa-mir-342 | hsa-miR-342-5p | -47.7 | 0.1451 | 0.0901 | 0.3397 | 2925 |
| hsa-mir-548e | hsa-miR-548e-5p | -39.3 | 0.1451 | 0.1734 | 0.2134 | 106 |
| hsa-mir-2355 | hsa-miR-2355-3p | -31.7 | 0.1443 | 0.2322 | 0.2486 | 203 |
| hsa-mir-320c-2 | hsa-miR-320c | -27.5 | 0.1442 | 0.1955 | 0.4106 | 1705 |
| hsa-mir-369 | hsa-miR-369-5p | -29.3 | 0.1441 | 0.0290 | 0.3073 | 107 |
| hsa-mir-1301 | hsa-miR-1301-3p | -45.8 | 0.1440 | 0.0915 | 0.4116 | 2868 |
| hsa-mir-181c | hsa-miR-181c-5p | -44.66 | 0.1431 | 0.0483 | 0.3294 | 1254 |
| hsa-mir-616 | hsa-miR-616-3p | -51.4 | 0.1430 | 0.1140 | 0.3626 | 124 |
| hsa-mir-369 | hsa-miR-369-3p | -29.3 | 0.1426 | 0.1737 | 0.2705 | 305 |
| hsa-mir-2277 | hsa-miR-2277-5p | -54.94 | 0.1423 | 0.0194 | 0.3469 | 91 |
| hsa-mir-4753 | hsa-miR-4753-5p | -46.4 | 0.1412 | 0.2037 | 0.2983 | 71 |
| hsa-mir-3150b | hsa-miR-3150b-3p | -70.3 | 0.1411 | 0.1308 | 0.3233 | 2723 |
| hsa-mir-361 | hsa-miR-361-3p | -32.3 | 0.1411 | 0.0548 | 0.4120 | 3646 |
| hsa-mir-106b | hsa-miR-106b-3p | -44.9 | 0.1410 | 0.1132 | 0.3481 | 2946 |
| hsa-mir-125a | hsa-miR-125a-3p | -48.1 | 0.1409 | 0.1490 | 0.2910 | 384 |
| hsa-mir-136 | hsa-miR-136-3p | -45.8 | 0.1395 | 0.1740 | 0.2802 | 243 |
| hsa-mir-202 | hsa-miR-202-5p | -58.8 | 0.1390 | 0.0845 | 0.2454 | 204 |
| hsa-mir-378c | hsa-miR-378c | -38.4 | 0.1389 | 0.0922 | 0.0916 | 2859 |
| hsa-mir-4476 | hsa-miR-4476 | -43.1 | 0.1389 | 0.3286 | 0.4715 | 512 |
| hsa-mir-516b-2 | hsa-miR-516b-5p | -37.3 | 0.1375 | 0.1248 | 0.2724 | 92 |
| hsa-mir-3138 | hsa-miR-3138 | -59.9 | 0.1368 | 0.3597 | 0.3498 | 202 |
| hsa-mir-744 | hsa-miR-744-5p | -38.9 | 0.1365 | 0.1271 | 0.4938 | 1607 |
| hsa-mir-627 | hsa-miR-627-5p | -56.5 | 0.1357 | 0.0895 | 0.2874 | 69 |
| hsa-mir-3614 | hsa-miR-3614-3p | -42.8 | 0.1357 | 0.1288 | 0.2904 | 58 |
| hsa-mir-18a | hsa-miR-18a-3p | -22 | 0.1355 | 0.0288 | 0.3698 | 229 |
| hsa-mir-204 | hsa-miR-204-5p | -42.26 | 0.1350 | 0.0992 | 0.2527 | 1432 |
| hsa-mir-1180 | hsa-miR-1180-3p | -39 | 0.1350 | 0.0294 | 0.3686 | 350 |
| hsa-mir-223 | hsa-miR-223-5p | -47.9 | 0.1346 | 0.0221 | 0.3933 | 1239 |
| hsa-mir-138-1 | hsa-miR-138-1-3p | -58.8 | 0.1340 | 0.2742 | 0.1669 | 171 |
| hsa-mir-34c | hsa-miR-34c-3p | -30 | 0.1338 | 0.1388 | 0.3073 | 136 |
| hsa-mir-188 | hsa-miR-188-5p | -39.8 | 0.1333 | 0.0450 | 0.3277 | 257 |
| hsa-mir-454 | hsa-miR-454-3p | -41.7 | 0.1329 | 0.0648 | 0.3515 | 891 |
| hsa-mir-378a | hsa-miR-378a-3p | -39.2 | 0.1321 | 0.1765 | 0.2499 | 6918 |
| hsa-mir-6514 | hsa-miR-6514-5p | -26.1 | 0.1319 | 0.1833 | 0.3150 | 75 |
| hsa-mir-299 | hsa-miR-299-3p | -40.5 | 0.1315 | 0.0685 | 0.3583 | 146 |
| hsa-let-7a-1 | hsa-let-7a-3p | -35.6 | 0.1308 | 0.0888 | 0.3257 | 799 |
| hsa-mir-3190 | hsa-miR-3190-3p | -55.5 | 0.1305 | 0.1331 | 0.2432 | 83 |
| hsa-mir-374b | hsa-miR-374b-3p | -41.4 | 0.1304 | 0.1122 | 0.3161 | 343 |
| hsa-let-7a-3 | hsa-let-7a-3p | -34.4 | 0.1301 | 0.0864 | 0.3258 | 798 |
| hsa-mir-132 | hsa-miR-132-3p | -48.4 | 0.1299 | 0.1523 | 0.2550 | 332 |
| hsa-mir-148b | hsa-miR-148b-5p | -35 | 0.1288 | 0.0650 | 0.2786 | 518 |
| hsa-mir-642a | hsa-miR-642a-5p | -65.4 | 0.1285 | 0.0000 | 0.2999 | 104 |
| hsa-mir-542 | hsa-miR-542-3p | -35.9 | 0.1277 | 0.1110 | 0.3028 | 2004 |
| hsa-mir-3940 | hsa-miR-3940-3p | -46.8 | 0.1276 | 0.0327 | 0.3705 | 77 |
| hsa-mir-665 | hsa-miR-665 | -34.3 | 0.1275 | 0.0086 | 0.6998 | 106 |
| hsa-mir-887 | hsa-miR-887-3p | -40.9 | 0.1272 | 0.0238 | 0.3989 | 1104 |
| hsa-mir-18b | hsa-miR-18b-3p | -19.8 | 0.1271 | 0.0359 | 0.3148 | 102 |
| hsa-mir-4724 | hsa-miR-4724-5p | -32.61 | 0.1270 | 0.0508 | 0.4147 | 54 |
| hsa-mir-377 | hsa-miR-377-3p | -27.8 | 0.1269 | 0.1424 | 0.2370 | 233 |
| hsa-mir-183 | hsa-miR-183-3p | -41.9 | 0.1257 | 0.0794 | 0.3016 | 135 |
| hsa-mir-9-1 | hsa-miR-9-3p | -42 | 0.1255 | 0.1445 | 0.2507 | 1231 |
| hsa-mir-378a | hsa-miR-378a-5p | -39.2 | 0.1254 | 0.1015 | 0.2006 | 266 |
| hsa-mir-501 | hsa-miR-501-3p | -43.1 | 0.1250 | 0.0555 | 0.3547 | 1098 |
| hsa-mir-9-2 | hsa-miR-9-3p | -40.1 | 0.1250 | 0.1434 | 0.2500 | 1230 |
| hsa-mir-16-2 | hsa-miR-16-2-3p | -30.8 | 0.1250 | 0.1225 | 0.2716 | 1741 |
| hsa-mir-337 | hsa-miR-337-3p | -40.3 | 0.1248 | 0.1530 | 0.2484 | 824 |
| hsa-mir-548t | hsa-miR-548t-5p | -40.2 | 0.1247 | 0.1259 | 0.1928 | 70 |
| hsa-mir-767 | hsa-miR-767-5p | -44.9 | 0.1236 | 0.0261 | 0.2349 | 652 |
| hsa-mir-30c-1 | hsa-miR-30c-1-3p | -35.4 | 0.1231 | 0.1020 | 0.2867 | 964 |
| hsa-mir-889 | hsa-miR-889-3p | -24.4 | 0.1230 | 0.0461 | 0.3456 | 219 |
| hsa-mir-92b | hsa-miR-92b-5p | -66.12 | 0.1229 | 0.0079 | 0.3589 | 3697 |
| hsa-mir-7-2 | hsa-miR-7-5p | -46.9 | 0.1222 | 0.0274 | 0.2807 | 8701 |
| hsa-mir-514a-1 | hsa-miR-514a-5p | -33.3 | 0.1221 | 0.0049 | 0.2937 | 1046 |
| hsa-mir-514a-2 | hsa-miR-514a-5p | -31.8 | 0.1221 | 0.0049 | 0.2937 | 1046 |
| hsa-mir-514a-3 | hsa-miR-514a-5p | -31.8 | 0.1221 | 0.0049 | 0.2937 | 1046 |
| hsa-mir-424 | hsa-miR-424-3p | -42.3 | 0.1220 | 0.0810 | 0.3050 | 1828 |
| hsa-mir-671 | hsa-miR-671-3p | -68.1 | 0.1219 | 0.0534 | 0.3286 | 161 |
| hsa-mir-3187 | hsa-miR-3187-3p | -44.7 | 0.1218 | 0.0243 | 0.3547 | 69 |
| hsa-mir-330 | hsa-miR-330-5p | -46.52 | 0.1210 | 0.0000 | 0.3361 | 90 |
| hsa-mir-200b | hsa-miR-200b-5p | -44.3 | 0.1205 | 0.0967 | 0.1904 | 281 |
| hsa-mir-106a | hsa-miR-106a-3p | -34.7 | 0.1198 | 0.1204 | 0.2504 | 93 |
| hsa-mir-1285-1 | hsa-miR-1285-3p | -58.2 | 0.1197 | 0.0205 | 0.3703 | 831 |
| hsa-mir-660 | hsa-miR-660-3p | -33.1 | 0.1196 | 0.0367 | 0.3451 | 299 |
| hsa-mir-190a | hsa-miR-190a-3p | -32.5 | 0.1195 | 0.1285 | 0.2394 | 106 |
| hsa-mir-302a | hsa-miR-302a-5p | -33.4 | 0.1194 | 0.0474 | 0.2395 | 647 |
| hsa-mir-1179 | hsa-miR-1179 | -33.6 | 0.1193 | 0.0813 | 0.2103 | 56 |
| hsa-mir-3609 | hsa-miR-3609 | -25.2 | 0.1190 | 0.1397 | 0.1963 | 225 |
| hsa-mir-7-1 | hsa-miR-7-5p | -48 | 0.1184 | 0.0268 | 0.2977 | 8973 |
| hsa-mir-548e | hsa-miR-548e-3p | -39.3 | 0.1183 | 0.1248 | 0.2674 | 558 |
| hsa-mir-146a | hsa-miR-146a-3p | -40.3 | 0.1179 | 0.1494 | 0.2436 | 181 |
| hsa-mir-32 | hsa-miR-32-3p | -31.9 | 0.1176 | 0.1481 | 0.2306 | 231 |
| hsa-mir-140 | hsa-miR-140-5p | -55 | 0.1172 | 0.1100 | 0.1713 | 544 |
| hsa-mir-629 | hsa-miR-629-3p | -74.8 | 0.1165 | 0.1030 | 0.2774 | 234 |
| hsa-mir-3614 | hsa-miR-3614-5p | -42.8 | 0.1164 | 0.0589 | 0.2387 | 288 |
| hsa-mir-642a | hsa-miR-642a-3p | -65.4 | 0.1162 | 0.0810 | 0.2912 | 183 |
| hsa-mir-9-3 | hsa-miR-9-3p | -41.1 | 0.1162 | 0.1479 | 0.2145 | 1131 |
| hsa-mir-411 | hsa-miR-411-5p | -30.7 | 0.1161 | 0.1174 | 0.1665 | 456 |
| hsa-mir-582 | hsa-miR-582-3p | -44.04 | 0.1160 | 0.0386 | 0.3482 | 689 |
| hsa-mir-138-2 | hsa-miR-138-5p | -36 | 0.1155 | 0.0374 | 0.3548 | 4133 |
| hsa-mir-379 | hsa-miR-379-5p | -26.6 | 0.1153 | 0.1431 | 0.1773 | 118 |
| hsa-mir-576 | hsa-miR-576-3p | -46.6 | 0.1148 | 0.0716 | 0.3027 | 478 |
| hsa-mir-320b-1 | hsa-miR-320b | -28 | 0.1137 | 0.1318 | 0.3997 | 6107 |
| hsa-mir-624 | hsa-miR-624-5p | -56.9 | 0.1137 | 0.0426 | 0.2607 | 80 |
| hsa-mir-3124 | hsa-miR-3124-5p | -32.5 | 0.1136 | 0.0250 | 0.2526 | 133 |
| hsa-mir-34a | hsa-miR-34a-3p | -50.7 | 0.1136 | 0.1305 | 0.2480 | 255 |
| hsa-mir-3200 | hsa-miR-3200-3p | -34.5 | 0.1129 | 0.0348 | 0.3415 | 65 |
| hsa-let-7i | hsa-let-7i-3p | -39.6 | 0.1126 | 0.0445 | 0.3082 | 587 |
| hsa-mir-365b | hsa-miR-365b-5p | -40.1 | 0.1125 | 0.0922 | 0.2203 | 2622 |
| hsa-mir-493 | hsa-miR-493-3p | -47.7 | 0.1122 | 0.0223 | 0.3185 | 178 |
| hsa-mir-454 | hsa-miR-454-5p | -41.7 | 0.1122 | 0.0000 | 0.3242 | 65 |
| hsa-mir-33a | hsa-miR-33a-3p | -37.3 | 0.1121 | 0.1266 | 0.2260 | 177 |
| hsa-mir-124-3 | hsa-miR-124-3p | -40.3 | 0.1121 | 0.0543 | 0.2917 | 415 |
| hsa-mir-3934 | hsa-miR-3934-5p | -43.7 | 0.1121 | 0.0306 | 0.2681 | 1194 |
| hsa-mir-340 | hsa-miR-340-3p | -36 | 0.1120 | 0.0874 | 0.2764 | 92 |
| hsa-mir-148b | hsa-miR-148b-3p | -35 | 0.1118 | 0.0595 | 0.2862 | 3176 |
| hsa-mir-30d | hsa-miR-30d-3p | -28.1 | 0.1118 | 0.0611 | 0.3117 | 225 |
| hsa-mir-450a-2 | hsa-miR-450a-2-3p | -35.4 | 0.1115 | 0.1250 | 0.2354 | 349 |
| hsa-mir-450b | hsa-miR-450b-5p | -23.5 | 0.1115 | 0.0605 | 0.1942 | 440 |
| hsa-mir-128-1 | hsa-miR-128-1-5p | -35.9 | 0.1112 | 0.0000 | 0.2471 | 59 |
| hsa-mir-1185-2 | hsa-miR-1185-2-3p | -38.5 | 0.1108 | 0.0214 | 0.3316 | 281 |
| hsa-mir-6721 | hsa-miR-6721-5p | -30.1 | 0.1108 | 0.0119 | 0.2097 | 142 |
| hsa-mir-576 | hsa-miR-576-5p | -46.6 | 0.1105 | 0.0117 | 0.2857 | 853 |
| hsa-mir-23b | hsa-miR-23b-5p | -36.04 | 0.1103 | 0.1495 | 0.1441 | 2887 |
| hsa-mir-1273c | hsa-miR-1273c | -46.1 | 0.1103 | 0.0000 | 0.2083 | 227 |
| hsa-mir-124-1 | hsa-miR-124-3p | -35.92 | 0.1102 | 0.0544 | 0.2857 | 414 |
| hsa-mir-124-2 | hsa-miR-124-3p | -51.2 | 0.1102 | 0.0544 | 0.2857 | 414 |
| hsa-mir-664a | hsa-miR-664a-3p | -24.4 | 0.1102 | 0.0613 | 0.2909 | 2592 |
| hsa-mir-3117 | hsa-miR-3117-3p | -29.3 | 0.1101 | 0.0529 | 0.3019 | 60 |
| hsa-mir-320e | hsa-miR-320e | -30.8 | 0.1099 | 0.0943 | 0.4188 | 370 |
| hsa-mir-495 | hsa-miR-495-3p | -35.6 | 0.1097 | 0.1238 | 0.2383 | 1199 |
| hsa-mir-153-2 | hsa-miR-153-3p | -41.3 | 0.1096 | 0.0336 | 0.3176 | 134 |
| hsa-mir-181c | hsa-miR-181c-3p | -44.66 | 0.1094 | 0.0681 | 0.2798 | 178 |
| hsa-mir-508 | hsa-miR-508-5p | -49.8 | 0.1092 | 0.0000 | 0.2426 | 3087 |
| hsa-mir-510 | hsa-miR-510-5p | -29.6 | 0.1092 | 0.0000 | 0.2790 | 336 |
| hsa-mir-545 | hsa-miR-545-3p | -37.52 | 0.1089 | 0.1027 | 0.2603 | 101 |
| hsa-mir-651 | hsa-miR-651-5p | -34.5 | 0.1088 | 0.0331 | 0.2514 | 348 |
| hsa-mir-21 | hsa-miR-21-3p | -35.8 | 0.1084 | 0.0626 | 0.2745 | 3944 |
| hsa-mir-141 | hsa-miR-141-5p | -48.9 | 0.1084 | 0.1041 | 0.2332 | 110 |
| hsa-mir-3180-1 | hsa-miR-3180-3p | -60.7 | 0.1083 | 0.0105 | 0.3505 | 1093 |
| hsa-mir-3180-2 | hsa-miR-3180-3p | -59.5 | 0.1083 | 0.0105 | 0.3505 | 1093 |
| hsa-mir-3180-3 | hsa-miR-3180-3p | -60.7 | 0.1083 | 0.0105 | 0.3505 | 1093 |
| hsa-mir-589 | hsa-miR-589-3p | -41.4 | 0.1079 | 0.1061 | 0.2730 | 224 |
| hsa-mir-4489 | hsa-miR-4489 | -31.8 | 0.1078 | 0.0471 | 0.1087 | 74 |
| hsa-mir-3136 | hsa-miR-3136-5p | -40.4 | 0.1078 | 0.0406 | 0.2469 | 129 |
| hsa-mir-4746 | hsa-miR-4746-5p | -34.9 | 0.1078 | 0.0516 | 0.1631 | 64 |
| hsa-mir-376b | hsa-miR-376b-3p | -34 | 0.1076 | 0.0558 | 0.3003 | 460 |
| hsa-mir-487b | hsa-miR-487b-3p | -34.8 | 0.1073 | 0.1277 | 0.2141 | 674 |
| hsa-mir-561 | hsa-miR-561-5p | -57.6 | 0.1068 | 0.0478 | 0.2015 | 122 |
| hsa-mir-500b | hsa-miR-500b-5p | -40.8 | 0.1068 | 0.0798 | 0.2761 | 155 |
| hsa-mir-1271 | hsa-miR-1271-5p | -50.2 | 0.1067 | 0.0106 | 0.2450 | 877 |
| hsa-mir-196b | hsa-miR-196b-3p | -34.3 | 0.1066 | 0.1248 | 0.2050 | 65 |
| hsa-mir-1276 | hsa-miR-1276 | -32.3 | 0.1060 | 0.0000 | 0.2239 | 177 |
| hsa-mir-1228 | hsa-miR-1228-5p | -46 | 0.1059 | 0.0857 | 0.3472 | 57 |
| hsa-mir-335 | hsa-miR-335-3p | -41.5 | 0.1058 | 0.1070 | 0.2226 | 318 |
| hsa-mir-345 | hsa-miR-345-5p | -51.3 | 0.1057 | 0.0110 | 0.2368 | 1176 |
| hsa-mir-125b-1 | hsa-miR-125b-1-3p | -43.4 | 0.1056 | 0.1157 | 0.2277 | 205 |
| hsa-mir-1323 | hsa-miR-1323 | -37.6 | 0.1051 | 0.0000 | 0.2101 | 198 |
| hsa-mir-1303 | hsa-miR-1303 | -43.9 | 0.1047 | 0.1542 | 0.4392 | 311 |
| hsa-mir-429 | hsa-miR-429 | -40.3 | 0.1046 | 0.2355 | 0.3352 | 226 |
| hsa-mir-3656 | hsa-miR-3656 | -31.5 | 0.1037 | 0.3133 | 0.3321 | 253 |
| hsa-mir-3613 | hsa-miR-3613-3p | -27.6 | 0.1035 | 0.0262 | 0.2973 | 283 |
| hsa-mir-628 | hsa-miR-628-5p | -36.4 | 0.1034 | 0.0307 | 0.2415 | 299 |
| hsa-let-7f-1 | hsa-let-7f-1-3p | -43.3 | 0.1031 | 0.1061 | 0.2300 | 409 |
| hsa-mir-147b | hsa-miR-147b | -28.5 | 0.1030 | 0.1370 | 0.4560 | 381 |
| hsa-mir-3653 | hsa-miR-3653 | -45.64 | 0.1028 | 0.5945 | 0.2779 | 54 |
| hsa-mir-514b | hsa-miR-514b-5p | -38 | 0.1028 | 0.0145 | 0.2482 | 259 |
| hsa-mir-493 | hsa-miR-493-5p | -47.7 | 0.1025 | 0.0538 | 0.2459 | 720 |
| hsa-mir-7-1 | hsa-miR-7-1-3p | -48 | 0.1023 | 0.1009 | 0.2380 | 1131 |
| hsa-mir-139 | hsa-miR-139-3p | -34.9 | 0.1022 | 0.0166 | 0.3355 | 93 |
| hsa-mir-378d-1 | hsa-miR-378d | -20.3 | 0.1022 | 0.1096 | 0.3219 | 146 |
| hsa-mir-550a-1 | hsa-miR-550a-3p | -46 | 0.1020 | 0.0670 | 0.2536 | 210 |
| hsa-mir-550a-2 | hsa-miR-550a-3p | -53.1 | 0.1020 | 0.0670 | 0.2536 | 210 |
| hsa-mir-550a-3 | hsa-miR-550a-3p | -43.9 | 0.1020 | 0.0670 | 0.2536 | 210 |
| hsa-mir-135b | hsa-miR-135b-3p | -47.2 | 0.1019 | 0.1257 | 0.1675 | 51 |
| hsa-mir-320c-1 | hsa-miR-320c | -34.67 | 0.1014 | 0.1912 | 0.4484 | 1825 |
| hsa-mir-181a-2 | hsa-miR-181a-2-3p | -54 | 0.1011 | 0.0437 | 0.2928 | 7480 |
| hsa-mir-224 | hsa-miR-224-5p | -36.6 | 0.1007 | 0.0315 | 0.3192 | 1480 |
| hsa-let-7d | hsa-let-7d-3p | -42.6 | 0.1003 | 0.1166 | 0.2029 | 601 |
| hsa-mir-1185-1 | hsa-miR-1185-5p | -35 | 0.1003 | 0.0000 | 0.2656 | 64 |
| hsa-mir-1185-2 | hsa-miR-1185-5p | -38.5 | 0.1003 | 0.0000 | 0.2656 | 64 |
| hsa-mir-26b | hsa-miR-26b-3p | -40.1 | 0.1002 | 0.0731 | 0.2476 | 233 |
| hsa-let-7e | hsa-let-7e-3p | -37.8 | 0.0998 | 0.0769 | 0.2428 | 149 |
| hsa-mir-1255b-2 | hsa-miR-1255b-5p | -40.6 | 0.0997 | 0.0481 | 0.1703 | 352 |
| hsa-mir-4436a | hsa-miR-4436a | -54.1 | 0.0992 | 0.3189 | 0.3331 | 54 |
| hsa-mir-769 | hsa-miR-769-5p | -60.1 | 0.0989 | 0.0259 | 0.2428 | 571 |
| hsa-mir-769 | hsa-miR-769-3p | -60.1 | 0.0983 | 0.0980 | 0.2345 | 163 |
| hsa-mir-516b-1 | hsa-miR-516b-5p | -44.9 | 0.0981 | 0.0547 | 0.2833 | 108 |
| hsa-mir-1306 | hsa-miR-1306-5p | -33.8 | 0.0975 | 0.0206 | 0.2577 | 318 |
| hsa-mir-504 | hsa-miR-504-5p | -52.3 | 0.0975 | 0.0736 | 0.1755 | 151 |
| hsa-mir-197 | hsa-miR-197-3p | -42.4 | 0.0971 | 0.0399 | 0.2702 | 1015 |
| hsa-mir-181a-1 | hsa-miR-181a-3p | -38.3 | 0.0968 | 0.0701 | 0.2522 | 9060 |
| hsa-mir-625 | hsa-miR-625-5p | -74.9 | 0.0964 | 0.0045 | 0.2484 | 1019 |
| hsa-mir-30c-2 | hsa-miR-30c-2-3p | -25.24 | 0.0963 | 0.0374 | 0.2801 | 2182 |
| hsa-mir-4484 | hsa-miR-4484 | -39.5 | 0.0957 | 0.3689 | 0.3329 | 291 |
| hsa-mir-296 | hsa-miR-296-5p | -41.4 | 0.0955 | 0.0780 | 0.1449 | 447 |
| hsa-mir-3127 | hsa-miR-3127-5p | -46.6 | 0.0953 | 0.0471 | 0.1753 | 287 |
| hsa-mir-302a | hsa-miR-302a-3p | -33.4 | 0.0943 | 0.0325 | 0.2908 | 4042 |
| hsa-mir-1468 | hsa-miR-1468-5p | -32 | 0.0942 | 0.0000 | 0.3036 | 88 |
| hsa-mir-193b | hsa-miR-193b-5p | -43.1 | 0.0941 | 0.0482 | 0.1883 | 4398 |
| hsa-mir-4437 | hsa-miR-4437 | -34.7 | 0.0940 | 0.2509 | 0.2399 | 70 |
| hsa-mir-15b | hsa-miR-15b-3p | -32.7 | 0.0937 | 0.0159 | 0.2895 | 1603 |
| hsa-mir-329-1 | hsa-miR-329-3p | -32.6 | 0.0937 | 0.0598 | 0.2454 | 268 |
| hsa-mir-329-2 | hsa-miR-329-3p | -35.2 | 0.0937 | 0.0598 | 0.2454 | 268 |
| hsa-mir-513b | hsa-miR-513b-5p | -35.7 | 0.0931 | 0.0000 | 0.2276 | 320 |
| hsa-mir-181d | hsa-miR-181d-5p | -69.1 | 0.0930 | 0.0074 | 0.2755 | 3137 |
| hsa-mir-4420 | hsa-miR-4420 | -29.4 | 0.0925 | 0.0984 | 0.5289 | 56 |
| hsa-mir-3690-2 | hsa-miR-3690 | -32.8 | 0.0908 | 0.0107 | 0.1609 | 81 |
| hsa-mir-625 | hsa-miR-625-3p | -74.9 | 0.0907 | 0.1157 | 0.1770 | 713 |
| hsa-mir-1287 | hsa-miR-1287-5p | -33.5 | 0.0893 | 0.0000 | 0.2447 | 626 |
| hsa-mir-16-1 | hsa-miR-16-1-3p | -37.7 | 0.0893 | 0.0977 | 0.2000 | 77 |
| hsa-mir-1185-1 | hsa-miR-1185-1-3p | -35 | 0.0892 | 0.0241 | 0.2657 | 695 |
| hsa-mir-499a | hsa-miR-499a-5p | -62.32 | 0.0888 | 0.0371 | 0.1846 | 743 |
| hsa-mir-1307 | hsa-miR-1307-5p | -57.3 | 0.0886 | 0.0089 | 0.2453 | 3342 |
| hsa-mir-1224 | hsa-miR-1224-5p | -45 | 0.0882 | 0.0523 | 0.4282 | 63 |
| hsa-mir-509-1 | hsa-miR-509-5p | -40.9 | 0.0874 | 0.0073 | 0.2063 | 8536 |
| hsa-mir-509-2 | hsa-miR-509-5p | -37.3 | 0.0874 | 0.0073 | 0.2063 | 8536 |
| hsa-mir-146b | hsa-miR-146b-3p | -37.1 | 0.0869 | 0.1347 | 0.1550 | 77 |
| hsa-mir-659 | hsa-miR-659-5p | -55 | 0.0869 | 0.0747 | 0.1528 | 267 |
| hsa-mir-664b | hsa-miR-664b-3p | -26.1 | 0.0862 | 0.0302 | 0.2573 | 125 |
| hsa-mir-206 | hsa-miR-206 | -43.9 | 0.0862 | 0.0653 | 0.4480 | 214 |
| hsa-mir-485 | hsa-miR-485-5p | -34.4 | 0.0859 | 0.0000 | 0.2196 | 637 |
| hsa-mir-877 | hsa-miR-877-5p | -39.5 | 0.0858 | 0.0780 | 0.4270 | 2681 |
| hsa-mir-514a-1 | hsa-miR-514a-3p | -33.3 | 0.0858 | 0.2458 | 0.0296 | 1360 |
| hsa-mir-514a-2 | hsa-miR-514a-3p | -31.8 | 0.0858 | 0.2458 | 0.0296 | 1360 |
| hsa-mir-514a-3 | hsa-miR-514a-3p | -31.8 | 0.0858 | 0.2458 | 0.0296 | 1360 |
| hsa-mir-22 | hsa-miR-22-5p | -39.8 | 0.0857 | 0.0190 | 0.2026 | 2281 |
| hsa-mir-1908 | hsa-miR-1908-5p | -45.2 | 0.0852 | 0.0369 | 0.1902 | 212 |
| hsa-mir-1306 | hsa-miR-1306-3p | -33.8 | 0.0849 | 0.0879 | 0.1574 | 405 |
| hsa-mir-151b | hsa-miR-151b | -30.7 | 0.0837 | 0.1342 | 0.4892 | 1222 |
| hsa-mir-2355 | hsa-miR-2355-5p | -31.7 | 0.0837 | 0.0133 | 0.2657 | 233 |
| hsa-let-7b | hsa-let-7b-3p | -50.6 | 0.0831 | 0.0473 | 0.2171 | 531 |
| hsa-mir-765 | hsa-miR-765 | -33 | 0.0828 | 0.2723 | 0.3756 | 105 |
| hsa-mir-513a-1 | hsa-miR-513a-5p | -62.9 | 0.0827 | 0.0000 | 0.2296 | 797 |
| hsa-mir-513a-2 | hsa-miR-513a-5p | -58.6 | 0.0827 | 0.0000 | 0.2296 | 797 |
| hsa-mir-548j | hsa-miR-548j-5p | -53.7 | 0.0821 | 0.0924 | 0.1083 | 410 |
| hsa-mir-1256 | hsa-miR-1256 | -50.22 | 0.0819 | 0.1419 | 0.2404 | 52 |
| hsa-mir-873 | hsa-miR-873-3p | -31.1 | 0.0817 | 0.1448 | 0.1275 | 260 |
| hsa-mir-2682 | hsa-miR-2682-5p | -46.9 | 0.0810 | 0.1030 | 0.0950 | 52 |
| hsa-mir-7-3 | hsa-miR-7-5p | -39.1 | 0.0809 | 0.0267 | 0.2836 | 8723 |
| hsa-mir-488 | hsa-miR-488-3p | -32.6 | 0.0806 | 0.0375 | 0.2222 | 96 |
| hsa-mir-432 | hsa-miR-432-5p | -42.1 | 0.0805 | 0.0169 | 0.2759 | 1149 |
| hsa-mir-551b | hsa-miR-551b-3p | -40 | 0.0802 | 0.0255 | 0.2330 | 126 |
| hsa-mir-203a | hsa-miR-203a | -56.4 | 0.0799 | 0.1997 | 0.3839 | 2445 |
| hsa-mir-940 | hsa-miR-940 | -54 | 0.0798 | 0.0604 | 0.4908 | 373 |
| hsa-mir-3918 | hsa-miR-3918 | -49.2 | 0.0797 | 0.0684 | 0.1619 | 92 |
| hsa-mir-150 | hsa-miR-150-3p | -57.5 | 0.0796 | 0.1012 | 0.1642 | 3504 |
| hsa-mir-1247 | hsa-miR-1247-3p | -57.7 | 0.0795 | 0.0240 | 0.1698 | 167 |
| hsa-mir-362 | hsa-miR-362-3p | -31.7 | 0.0794 | 0.0382 | 0.2059 | 972 |
| hsa-mir-4446 | hsa-miR-4446-3p | -28.22 | 0.0791 | 0.0640 | 0.1995 | 241 |
| hsa-mir-4520b | hsa-miR-4520b-3p | -39.9 | 0.0786 | 0.1139 | 0.1482 | 67 |
| hsa-mir-383 | hsa-miR-383-5p | -27.3 | 0.0785 | 0.0733 | 0.1798 | 149 |
| hsa-mir-652 | hsa-miR-652-3p | -55.2 | 0.0785 | 0.0246 | 0.2223 | 2959 |
| hsa-mir-3611 | hsa-miR-3611 | -31.72 | 0.0784 | 0.0544 | 0.4243 | 120 |
| hsa-mir-4487 | hsa-miR-4487 | -62.1 | 0.0781 | 0.2675 | 0.2505 | 74 |
| hsa-mir-577 | hsa-miR-577 | -49.02 | 0.0780 | 0.0686 | 0.1307 | 724 |
| hsa-mir-129-1 | hsa-miR-129-1-3p | -29.4 | 0.0770 | 0.0756 | 0.1781 | 1141 |
| hsa-mir-513c | hsa-miR-513c-5p | -36.6 | 0.0768 | 0.0121 | 0.1671 | 784 |
| hsa-mir-33a | hsa-miR-33a-5p | -37.3 | 0.0760 | 0.0128 | 0.2009 | 3268 |
| hsa-mir-99b | hsa-miR-99b-3p | -30 | 0.0760 | 0.0024 | 0.2473 | 957 |
| hsa-let-7g | hsa-let-7g-3p | -40.5 | 0.0760 | 0.0144 | 0.2305 | 56 |
| hsa-mir-766 | hsa-miR-766-3p | -50.8 | 0.0757 | 0.0308 | 0.2216 | 1102 |
| hsa-mir-3180-4 | hsa-miR-3180 | -102.2 | 0.0756 | 0.0105 | 0.3505 | 1093 |
| hsa-mir-3180-5 | hsa-miR-3180 | -107.3 | 0.0756 | 0.0105 | 0.3505 | 1093 |
| hsa-mir-598 | hsa-miR-598-3p | -38.3 | 0.0754 | 0.0057 | 0.2455 | 2714 |
| hsa-mir-212 | hsa-miR-212-5p | -50.8 | 0.0753 | 0.0354 | 0.1738 | 336 |
| hsa-mir-4431 | hsa-miR-4431 | -46.9 | 0.0752 | 0.0274 | 0.0895 | 195 |
| hsa-mir-302c | hsa-miR-302c-3p | -32 | 0.0751 | 0.0081 | 0.2497 | 8444 |
| hsa-mir-3180-3 | hsa-miR-3180-5p | -60.7 | 0.0747 | 0.0138 | 0.1704 | 176 |
| hsa-mir-301b | hsa-miR-301b | -33 | 0.0743 | 0.0387 | 0.3821 | 239 |
| hsa-mir-34c | hsa-miR-34c-5p | -30 | 0.0734 | 0.0140 | 0.1257 | 8087 |
| hsa-mir-376a-2 | hsa-miR-376a-3p | -28.2 | 0.0725 | 0.0732 | 0.1511 | 349 |
| hsa-mir-27a | hsa-miR-27a-5p | -37.5 | 0.0714 | 0.0190 | 0.1903 | 963 |
| hsa-mir-98 | hsa-miR-98-3p | -57 | 0.0714 | 0.0750 | 0.1629 | 323 |
| hsa-mir-3145 | hsa-miR-3145-3p | -43.2 | 0.0710 | 0.1657 | 0.0868 | 58 |
| hsa-mir-3928 | hsa-miR-3928-3p | -40.5 | 0.0707 | 0.0732 | 0.1623 | 440 |
| hsa-mir-548s | hsa-miR-548s | -39.7 | 0.0706 | 0.1917 | 0.2714 | 79 |
| hsa-mir-1255a | hsa-miR-1255a | -61.3 | 0.0693 | 0.0196 | 0.2481 | 1558 |
| hsa-mir-449c | hsa-miR-449c-5p | -46.4 | 0.0691 | 0.0275 | 0.1387 | 194 |
| hsa-mir-548b | hsa-miR-548b-3p | -48.6 | 0.0690 | 0.0237 | 0.2064 | 71 |
| hsa-mir-320b-2 | hsa-miR-320b | -46.3 | 0.0688 | 0.1301 | 0.4258 | 6290 |
| hsa-mir-122 | hsa-miR-122-5p | -46.6 | 0.0688 | 0.0136 | 0.1546 | 895 |
| hsa-mir-376a-1 | hsa-miR-376a-3p | -18.2 | 0.0684 | 0.0721 | 0.1420 | 348 |
| hsa-mir-548k | hsa-miR-548k | -46 | 0.0681 | 0.0170 | 0.2745 | 1038 |
| hsa-mir-1277 | hsa-miR-1277-3p | -34.6 | 0.0678 | 0.0074 | 0.2186 | 252 |
| hsa-mir-30b | hsa-miR-30b-3p | -37.6 | 0.0678 | 0.0168 | 0.2059 | 2214 |
| hsa-mir-758 | hsa-miR-758-3p | -29.4 | 0.0674 | 0.0340 | 0.1823 | 107 |
| hsa-mir-375 | hsa-miR-375 | -26.2 | 0.0674 | 0.0942 | 0.2494 | 565 |
| hsa-mir-544b | hsa-miR-544b | -23.4 | 0.0669 | 0.1616 | 0.2474 | 64 |
| hsa-mir-4421 | hsa-miR-4421 | -26.2 | 0.0669 | 0.1082 | 0.2632 | 399 |
| hsa-mir-532 | hsa-miR-532-3p | -25.72 | 0.0668 | 0.0586 | 0.1503 | 535 |
| hsa-mir-5187 | hsa-miR-5187-5p | -25.5 | 0.0665 | 0.0515 | 0.1552 | 232 |
| hsa-mir-4425 | hsa-miR-4425 | -54.3 | 0.0658 | 0.1584 | 0.2581 | 1042 |
| hsa-mir-589 | hsa-miR-589-5p | -41.4 | 0.0657 | 0.0043 | 0.1636 | 1359 |
| hsa-mir-506 | hsa-miR-506-5p | -44.76 | 0.0656 | 0.0084 | 0.1520 | 498 |
| hsa-mir-133b | hsa-miR-133b | -48.4 | 0.0653 | 0.0990 | 0.3599 | 327 |
| hsa-mir-3180-1 | hsa-miR-3180-5p | -60.7 | 0.0652 | 0.0139 | 0.1678 | 175 |
| hsa-mir-3180-2 | hsa-miR-3180-5p | -59.5 | 0.0652 | 0.0139 | 0.1678 | 175 |
| hsa-mir-148a | hsa-miR-148a-5p | -28.2 | 0.0651 | 0.0547 | 0.1189 | 1145 |
| hsa-let-7c | hsa-let-7c-3p | -33.5 | 0.0644 | 0.0508 | 0.1637 | 259 |
| hsa-mir-3158-1 | hsa-miR-3158-3p | -74.1 | 0.0643 | 0.0400 | 0.1742 | 180 |
| hsa-mir-219a-1 | hsa-miR-219a-1-3p | -54.5 | 0.0642 | 0.0264 | 0.1877 | 145 |
| hsa-mir-4524a | hsa-miR-4524a-3p | -38.24 | 0.0635 | 0.0235 | 0.1881 | 72 |
| hsa-mir-363 | hsa-miR-363-5p | -25.4 | 0.0625 | 0.0498 | 0.1501 | 2589 |
| hsa-mir-155 | hsa-miR-155-3p | -29.7 | 0.0617 | 0.0347 | 0.1529 | 162 |
| hsa-mir-1910 | hsa-miR-1910-3p | -34.2 | 0.0613 | 0.0000 | 0.1907 | 68 |
| hsa-mir-129-2 | hsa-miR-129-2-3p | -48.8 | 0.0610 | 0.0163 | 0.1864 | 2196 |
| hsa-mir-374a | hsa-miR-374a-3p | -35.2 | 0.0610 | 0.0158 | 0.1843 | 2977 |
| hsa-mir-1272 | hsa-miR-1272 | -43.3 | 0.0607 | 0.1821 | 0.0000 | 72 |
| hsa-mir-1294 | hsa-miR-1294 | -70.2 | 0.0606 | 0.0535 | 0.3168 | 165 |
| hsa-mir-513c | hsa-miR-513c-3p | -36.6 | 0.0606 | 0.1200 | 0.0885 | 253 |
| hsa-mir-4741 | hsa-miR-4741 | -54 | 0.0603 | 0.0783 | 0.3451 | 155 |
| hsa-mir-1249 | hsa-miR-1249 | -32.7 | 0.0603 | 0.0221 | 0.2994 | 250 |
| hsa-mir-3130-1 | hsa-miR-3130-3p | -71.2 | 0.0597 | 0.0483 | 0.1439 | 162 |
| hsa-mir-3130-2 | hsa-miR-3130-3p | -69.9 | 0.0597 | 0.0483 | 0.1439 | 162 |
| hsa-mir-185 | hsa-miR-185-3p | -53.1 | 0.0597 | 0.0550 | 0.1439 | 1088 |
| hsa-mir-549a | hsa-miR-549a | -53.2 | 0.0597 | 0.1478 | 0.3029 | 51 |
| hsa-mir-433 | hsa-miR-433-3p | -38.2 | 0.0593 | 0.0912 | 0.0958 | 727 |
| hsa-mir-944 | hsa-miR-944 | -45.6 | 0.0592 | 0.2312 | 0.1593 | 382 |
| hsa-mir-1284 | hsa-miR-1284 | -34.39 | 0.0590 | 0.0328 | 0.2096 | 96 |
| hsa-mir-345 | hsa-miR-345-3p | -51.3 | 0.0587 | 0.1217 | 0.0739 | 75 |
| hsa-mir-382 | hsa-miR-382-3p | -28.5 | 0.0586 | 0.0198 | 0.1689 | 1175 |
| hsa-mir-129-2 | hsa-miR-129-5p | -48.8 | 0.0584 | 0.0023 | 0.1860 | 3018 |
| hsa-mir-655 | hsa-miR-655-3p | -35.1 | 0.0581 | 0.0098 | 0.1810 | 403 |
| hsa-mir-23c | hsa-miR-23c | -33.4 | 0.0572 | 0.1151 | 0.2981 | 73 |
| hsa-mir-92b | hsa-miR-92b-3p | -66.12 | 0.0571 | 0.0273 | 0.1589 | 5957 |
| hsa-mir-643 | hsa-miR-643 | -45.9 | 0.0568 | 0.1586 | 0.2705 | 69 |
| hsa-mir-584 | hsa-miR-584-5p | -56 | 0.0563 | 0.0335 | 0.1220 | 7197 |
| hsa-mir-181b-1 | hsa-miR-181b-3p | -34.9 | 0.0559 | 0.0327 | 0.1476 | 757 |
| hsa-mir-5010 | hsa-miR-5010-5p | -44.2 | 0.0557 | 0.0356 | 0.2429 | 578 |
| hsa-mir-941-1 | hsa-miR-941 | -39.5 | 0.0557 | 0.0074 | 0.3266 | 126 |
| hsa-mir-941-4 | hsa-miR-941 | -43.1 | 0.0557 | 0.0074 | 0.3266 | 126 |
| hsa-mir-323a | hsa-miR-323a-3p | -37.7 | 0.0553 | 0.1141 | 0.0640 | 442 |
| hsa-mir-20b | hsa-miR-20b-5p | -29.8 | 0.0552 | 0.0163 | 0.1097 | 3383 |
| hsa-mir-211 | hsa-miR-211-3p | -45.8 | 0.0547 | 0.0225 | 0.1539 | 450 |
| hsa-mir-548o-2 | hsa-miR-548o-3p | -39.3 | 0.0541 | 0.0000 | 0.1802 | 146 |
| hsa-mir-129-1 | hsa-miR-129-5p | -29.4 | 0.0539 | 0.0009 | 0.1849 | 3013 |
| hsa-mir-153-1 | hsa-miR-153-3p | -48.1 | 0.0531 | 0.0284 | 0.3173 | 133 |
| hsa-mir-3158-2 | hsa-miR-3158-3p | -70.66 | 0.0525 | 0.0314 | 0.1436 | 180 |
| hsa-mir-411 | hsa-miR-411-3p | -30.7 | 0.0523 | 0.0048 | 0.1598 | 210 |
| hsa-mir-584 | hsa-miR-584-3p | -56 | 0.0516 | 0.0252 | 0.1468 | 191 |
| hsa-mir-513a-1 | hsa-miR-513a-3p | -62.9 | 0.0515 | 0.0804 | 0.0968 | 209 |
| hsa-mir-513a-2 | hsa-miR-513a-3p | -58.6 | 0.0515 | 0.0804 | 0.0968 | 209 |
| hsa-mir-3135a | hsa-miR-3135a | -33.3 | 0.0513 | 0.0000 | 0.0969 | 58 |
| hsa-mir-301a | hsa-miR-301a-5p | -32.8 | 0.0513 | 0.0068 | 0.1242 | 1123 |
| hsa-mir-3153 | hsa-miR-3153 | -47 | 0.0504 | 0.0870 | 0.2322 | 54 |
| hsa-mir-506 | hsa-miR-506-3p | -44.76 | 0.0503 | 0.1318 | 0.0304 | 490 |
| hsa-mir-1290 | hsa-miR-1290 | -23.3 | 0.0502 | 0.1607 | 0.1738 | 66 |
| hsa-mir-188 | hsa-miR-188-3p | -39.8 | 0.0501 | 0.0284 | 0.1330 | 93 |
| hsa-mir-3615 | hsa-miR-3615 | -45.8 | 0.0500 | 0.0038 | 0.3187 | 1015 |
| hsa-mir-137 | hsa-miR-137 | -49.1 | 0.0491 | 0.1198 | 0.2402 | 52 |
| hsa-mir-421 | hsa-miR-421 | -35.2 | 0.0486 | 0.1208 | 0.1667 | 1420 |
| hsa-mir-1295a | hsa-miR-1295a | -49.9 | 0.0482 | 0.0552 | 0.2241 | 196 |
| hsa-mir-1285-2 | hsa-miR-1285-3p | -31.7 | 0.0481 | 0.0198 | 0.2847 | 590 |
| hsa-mir-134 | hsa-miR-134-5p | -35.2 | 0.0479 | 0.0081 | 0.1124 | 1041 |
| hsa-mir-548a-3 | hsa-miR-548a-3p | -45.9 | 0.0475 | 0.0162 | 0.1423 | 272 |
| hsa-mir-548ac | hsa-miR-548ac | -50.5 | 0.0473 | 0.1939 | 0.1213 | 51 |
| hsa-mir-3175 | hsa-miR-3175 | -40.6 | 0.0470 | 0.0000 | 0.0888 | 103 |
| hsa-mir-486 | hsa-miR-486-3p | -48.5 | 0.0467 | 0.0134 | 0.1351 | 2637 |
| hsa-mir-615 | hsa-miR-615-5p | -58.8 | 0.0447 | 0.0132 | 0.1307 | 124 |
| hsa-mir-548u | hsa-miR-548u | -36.3 | 0.0427 | 0.0000 | 0.2654 | 78 |
| hsa-mir-1286 | hsa-miR-1286 | -32.3 | 0.0426 | 0.0000 | 0.2559 | 229 |
| hsa-mir-410 | hsa-miR-410-3p | -36.7 | 0.0426 | 0.0255 | 0.0989 | 151 |
| hsa-mir-3121 | hsa-miR-3121-3p | -39.9 | 0.0419 | 0.0446 | 0.0950 | 64 |
| hsa-mir-92a-2 | hsa-miR-92a-2-5p | -30.2 | 0.0416 | 0.0096 | 0.1058 | 450 |
| hsa-mir-378f | hsa-miR-378f | -22.09 | 0.0394 | 0.0377 | 0.2206 | 109 |
| hsa-mir-485 | hsa-miR-485-3p | -34.4 | 0.0394 | 0.0809 | 0.0503 | 561 |
| hsa-mir-551a | hsa-miR-551a | -55.3 | 0.0371 | 0.0482 | 0.2317 | 56 |
| hsa-mir-3179-1 | hsa-miR-3179 | -61 | 0.0360 | 0.0963 | 0.1396 | 187 |
| hsa-mir-760 | hsa-miR-760 | -41.6 | 0.0353 | 0.0368 | 0.1830 | 701 |
| hsa-mir-184 | hsa-miR-184 | -37.9 | 0.0342 | 0.0058 | 0.2194 | 1146 |
| hsa-mir-656 | hsa-miR-656-3p | -23.7 | 0.0328 | 0.0232 | 0.0824 | 73 |
| hsa-mir-3179-2 | hsa-miR-3179 | -61 | 0.0327 | 0.0962 | 0.1179 | 184 |
| hsa-mir-3179-3 | hsa-miR-3179 | -61 | 0.0327 | 0.0962 | 0.1179 | 184 |
| hsa-mir-1299 | hsa-miR-1299 | -34.34 | 0.0325 | 0.0137 | 0.2311 | 551 |
| hsa-mir-543 | hsa-miR-543 | -25.5 | 0.0324 | 0.0202 | 0.1696 | 1966 |
| hsa-mir-1537 | hsa-miR-1537-3p | -28.6 | 0.0318 | 0.0151 | 0.0909 | 105 |
| hsa-mir-1278 | hsa-miR-1278 | -50.2 | 0.0316 | 0.0365 | 0.1638 | 712 |
| hsa-mir-3909 | hsa-miR-3909 | -58.13 | 0.0305 | 0.0515 | 0.2130 | 90 |
| hsa-mir-1269a | hsa-miR-1269a | -70.61 | 0.0287 | 0.0237 | 0.2119 | 4347 |
| hsa-mir-3198-2 | hsa-miR-3198 | -44.5 | 0.0253 | 0.0144 | 0.1430 | 56 |
| hsa-mir-3689b | hsa-miR-3689b-5p | -81 | 0.0252 | 0.0108 | 0.0620 | 80 |
| hsa-mir-378e | hsa-miR-378e | -35.1 | 0.0247 | 0.0351 | 0.1433 | 105 |
| hsa-mir-3164 | hsa-miR-3164 | -46.3 | 0.0224 | 0.0000 | 0.0423 | 237 |
| hsa-mir-5196 | hsa-miR-5196-3p | -53.8 | 0.0216 | 0.0168 | 0.0527 | 177 |
| hsa-mir-3198-1 | hsa-miR-3198 | -50.2 | 0.0211 | 0.0000 | 0.1314 | 54 |
| hsa-mir-548a-2 | hsa-miR-548a-3p | -39.3 | 0.0210 | 0.0162 | 0.1423 | 272 |
| hsa-mir-3662 | hsa-miR-3662 | -19.6 | 0.0206 | 0.0785 | 0.0633 | 78 |
| hsa-mir-548a-1 | hsa-miR-548a-3p | -35.6 | 0.0195 | 0.0039 | 0.1437 | 268 |
| hsa-mir-380 | hsa-miR-380-3p | -24.8 | 0.0160 | 0.0000 | 0.0533 | 70 |
| hsa-mir-518b | hsa-miR-518b | -39.7 | 0.0148 | 0.0000 | 0.0957 | 56 |
| hsa-mir-4435-2 | hsa-miR-4435 | -26.3 | 0.0134 | 0.0000 | 0.0178 | 128 |
| hsa-mir-548o | hsa-miR-548o-3p | -22.6 | 0.0119 | 0.0000 | 0.1239 | 132 |
| hsa-mir-6720 | hsa-miR-6720-3p | -39.62 | 0.0103 | 0.0000 | 0.0343 | 181 |
| hsa-mir-4435-1 | hsa-miR-4435 | -30 | 0.0094 | 0.0000 | 0.0178 | 128 |
| hsa-mir-3123 | hsa-miR-3123 | -31.1 | 0.0069 | 0.0180 | 0.0247 | 100 |
| hsa-mir-941-2 | hsa-miR-941 | -43.1 | 0.0030 | 0.0091 | 0.0091 | 98 |
| hsa-mir-378b | hsa-miR-378b | -24.9 | 0.0030 | 0.0000 | 0.0135 | 831 |
