# Supplementary material for: Entropy-Based Model for MiRNA Isoform Analysis
Source: PLoS One. 2015 Mar 18;10(3):e0118856. doi: 10.1371/journal.pone.0118856 (PMC4364746; doi:10.1371/journal.pone.0118856)
Supplement: S2 Table — (DOC) [file pone.0118856.s003.doc]

S2 Table. The KEGG pathway enrichment of the experimentally validated miRNA target genes using the DAVID Bioinformatics Tools.

| Cluster | Enrichment Score | Term | Count | PValue | Benjamini |
| --- | --- | --- | --- | --- | --- |
| 1 | 18.29 | Pathways in cancer | 99 | 2.1E-38 | 3.1E-36 |
|  |  | Chronic myeloid leukemia | 39 | 2.0E-24 | 1.5E-22 |
|  |  | Prostate cancer | 41 | 3.8E-23 | 1.9E-21 |
|  |  | Pancreatic cancer | 37 | 6.4E-23 | 2.4E-21 |
|  |  | Bladder cancer | 24 | 3.6E-16 | 6.1E-15 |
|  |  | Glioma | 28 | 2.8E-15 | 4.5E-14 |
|  |  | Melanoma | 28 | 9.9E-14 | 1.2E-12 |
|  |  | Non-small cell lung cancer | 24 | 4.1E-13 | 4.6E-12 |
|  |  | Endometrial cancer | 23 | 1.6E-12 | 1.6E-11 |
|  |  | Acute myeloid leukemia | 22 | 1.8E-10 | 1.5E-09 |
| 2 | 8.42 | Glioma | 28 | 2.8E-15 | 4.5E-14 |
|  |  | Neurotrophin signaling pathway | 38 | 1.8E-14 | 2.4E-13 |
|  |  | Non-small cell lung cancer | 24 | 4.1E-13 | 4.6E-12 |
|  |  | Endometrial cancer | 23 | 1.6E-12 | 1.6E-11 |
|  |  | ErbB signaling pathway | 28 | 2.7E-11 | 2.3E-10 |
|  |  | Acute myeloid leukemia | 22 | 1.8E-10 | 1.5E-09 |
|  |  | Renal cell carcinoma | 24 | 2.3E-10 | 1.8E-09 |
|  |  | Insulin signaling pathway | 30 | 6.2E-08 | 4.1E-07 |
|  |  | VEGF signaling pathway | 20 | 9.0E-07 | 5.3E-06 |
|  |  | Fc epsilon RI signaling pathway | 20 | 1.7E-06 | 9.7E-06 |
|  |  | T cell receptor signaling pathway | 24 | 1.8E-06 | 9.6E-06 |
|  |  | B cell receptor signaling pathway | 18 | 1.7E-05 | 7.2E-05 |
|  |  | Chemokine signaling pathway | 28 | 3.6E-04 | 1.4E-03 |
|  |  | Fc gamma R-mediated phagocytosis | 16 | 3.1E-03 | 1.0E-02 |
