# Supplementary material for: Entropy-Based Model for MiRNA Isoform Analysis
Source: PLoS One. 2015 Mar 18;10(3):e0118856. doi: 10.1371/journal.pone.0118856 (PMC4364746; doi:10.1371/journal.pone.0118856)
Supplement: S3 Table — (DOC) [file pone.0118856.s004.doc]

S3 Table. The KEGG pathway enrichment of the TargetScan predicted miRNA target genes using the DAVID Bioinformatics Tools.

| Cluster | Enrichment Score | Term | Count | PValue | Benjamini |
| --- | --- | --- | --- | --- | --- |
| 1 | 2.94 | Melanoma | 15 | 3.2E-04 | 1.3E-02 |
|  |  | Chronic myeloid leukemia | 15 | 5.8E-04 | 1.5E-02 |
|  |  | Glioma | 13 | 1.2E-03 | 2.3E-02 |
|  |  | Bladder cancer | 9 | 7.7E-03 | 7.5E-02 |
| 2 | 2.37 | Colorectal cancer | 18 | 5.3E-05 | 2.8E-03 |
|  |  | Basal cell carcinoma | 10 | 1.3E-02 | 1.1E-01 |
|  |  | Wnt signaling pathway | 19 | 1.7E-02 | 1.2E-01 |
|  |  | Endometrial cancer | 9 | 2.7E-02 | 1.7E-01 |
| 3 | 2.29 | Hypertrophic cardiomyopathy (HCM) | 15 | 2.1E-03 | 3.0E-02 |
|  |  | Arrhythmogenic right ventricular cardiomyopathy (ARVC) | 13 | 6.0E-03 | 7.2E-02 |
|  |  | Dilated cardiomyopathy | 14 | 1.1E-02 | 9.5E-02 |
| 4 | 2.11 | Prostate cancer | 17 | 3.7E-04 | 1.2E-02 |
|  |  | Endometrial cancer | 9 | 2.7E-02 | 1.7E-01 |
|  |  | Acute myeloid leukemia | 9 | 4.7E-02 | 2.5E-01 |
